# The long non‐coding RNA DANCR regulates the inflammatory phenotype of breast cancer cells and promotes breast cancer progression *via* EZH2‐dependent suppression of SOCS3 transcription

**DOI:** 10.1002/1878-0261.12622

**Published:** 2020-01-10

**Authors:** Ke‐Jing Zhang, Xiao‐Lang Tan, Lei Guo

**Affiliations:** ^1^ Department of Breast Surgery Xiangya Hospital Clinical Research Center For Breast Cancer Control and Prevention in Hunan Province Central South University Changsha China; ^2^ Department of Oncology Changsha Central Hospital China

**Keywords:** breast cancer, DANCR, epigenetic modification, EZH2, interleukin ‐6, SOCS3

## Abstract

Long non‐coding RNA (lncRNA) is involved in the regulation of tumorigenesis and metastasis. In this study, we focused on the clinical relevance, biological effects, and molecular mechanisms of the lncRNA differentiation antagonizing non‐protein coding RNA (DANCR) in breast cancer. We compared the expression of DANCR between breast cancer and normal tissues, and between breast cancer cell lines and normal breast epithelial cells using quantitative real‐time PCR (qRT‐PCR) analysis. By knocking down and overexpressing DANCR, we assessed its significance in regulating viability (MTT assay), migration/invasion (Transwell assay), epithelial‐mesenchymal transition (western blot), stemness (mammosphere formation assay and western blot), and production of inflammatory cytokines (qRT‐PCR and ELISA) of breast cancer cells *in vitro*, as well as xenograft growth *in vivo*. Furthermore, using ChIP and RNA immunoprecipitation, we examined the reciprocal regulation between DANCR and suppressor of cytokine signaling 3 (SOCS3) in breast cancer. DANCR was significantly up‐regulated in tissue samples from patients with breast cancer, as well as in breast cancer cell lines, as compared with normal tissues and breast epithelial cells, respectively. The highest DANCR expression levels were associated with advanced tumor grades or lymph node metastasis. DANCR was necessary and sufficient to control multiple malignant phenotypes of breast cancer cells *in vitro* and xenograft growth *in vivo*. Mechanistically, DANCR promoted the binding of enhancer of zeste homolog 2 (EZH2) to the promoter of SOCS3, thereby epigenetically inhibiting SOCS3 expression. Functionally, SOCS3 up‐regulation or EZH2 inhibition could rescue multiple malignant phenotypes induced by DANCR. Our data indicate that DANCR is a pleiotropic oncogenic lncRNA in breast cancer. Boosting SOCS3 expression may reverse the oncogenic activities of DANCR and thus provide a therapeutic strategy for breast cancer treatment.

## Graphical abstract

Differentiation antagonizing non‐protein coding RNA (DANCR) binds to enhancer of zeste homolog 2 (EZH2), recruiting EZH2‐ and EZH2‐generated H3K27me3 to the suppressor of cytokine signaling 3 (SOCS3) promoter, thus inhibiting the transcription of SOCS3. DANCR induced down‐regulation of SOCS3‐induced inflammatory response and multiple malignant phenotypes in breast cancer cell.

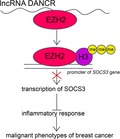

**Abbreviations**
ABCG2: ATP Binding Cassette Subfamily G Member 2
ALDH: aldehyde dehydrogenase
CSC: cancer stem cells
DANCR: differentiation antagonizing non‐protein coding RNA
DMEM: Dulbecco’s modified Eagle’s medium
EMT: epithelial‐mesenchymal transition
EZH2: enhancer of zeste homolog 2
IL‐6: interleukin 6
lncRNA: long non‐coding RNA
PI: propidium iodide
qRT‐PCR: quantitative real‐time PCR
RIP assay: RNA immunoprecipitation
sh: short‐hairpin RNA
SOCS3: suppressor of cytokine signaling 3
STAT3: signal transducer and activator of transcription 3
TCGA: The Cancer Genome Atlas
TGF‐β: transforming growth factor β
TNBC: triple‐negative breast cancer

## 1 Introduction

Breast cancer is a highly heterogeneous disease that remains the most common malignancy and the second leading cause of cancer‐related death (after lung cancer) in women worldwide (Torre *et al.*, 2015). Development in gene expression profiling classified breast cancer into five intrinsic subtypes associated with distinct clinical features and thus different treatment strategies. Of these subtypes, ~ 12.3% of total breast cancer cases are made up of the basal subtype, which is characterized by negative expressions of estrogen receptor, progesterone receptor, and human EGF receptor 2 [thus commonly referred to as triple‐negative breast cancer (TNBC)], and is associated with a high chance of metastasis and the poorest prognosis (Dai *et al.*, 2015). Overall, tumor metastasis is the major cause of death in breast cancer patients.

Many biological processes contributed to the malignant development and metastatic spread of breast cancer, such as epithelial‐mesenchymal transition (EMT) (Wang and Zhou, 2011), cancer stemness (Velasco‐Velazquez *et al.*, 2011), and dysregulated immune responses (Janssen *et al.*, 2017). Interestingly, these biological processes do not act alone but rather through intricate communications collectively, and sometimes synergistically stimulate the malignant progression of breast cancer (Reiman *et al.*, 2010). For example, overexpression of EMT‐related biomarkers such as Snail, Twist, and FoxC2 endows breast cancer cells with stem features (Hollier *et al.*, 2013; Mani *et al.*, 2008). Many signaling pathways regulating stem cell renewal and differentiation, such as transforming growth factor β (TGF‐β), Wnt, Notch, and Hedgehog, are also important EMT inducers (Wang *et al.*, 2015). Inflammation‐regulating cytokines such as TGF‐β and interleukin‐6 (IL‐6) are potent inducers of EMT and cancer stem cells (CSC) in breast cancer (Barcellos‐Hoff and Akhurst, 2009; Xie *et al.*, 2012). Therefore, understanding mechanisms controlling the crosstalk among EMT, cancer stemness, and inflammation will benefit the development of therapeutic options simultaneously targeting all three malignant phenotypes, and significantly improve the prognosis of breast cancer patients.

Long non‐coding RNA (lncRNA) is a class of non‐coding RNA molecules containing > 200 nucleotides and regulating gene expression through a variety of mechanisms, including chromatin remodeling, regulation of gene expression on transcriptional, post‐transcriptional, translational, and post‐translational level, modulation of other RNA species such as microRNA, and generation of small interfering RNA (Fang and Fullwood, 2016). Functionally, lncRNA critically regulates the pathogenesis of human diseases, including tumorigenesis and cancer progression (Chen *et al.*, 2017; Dou *et al.*, 2017; Qian *et al.*, 2017; Wang *et al.*, 2017; Zuo *et al.*, 2017). The differentiation antagonizing non‐protein coding RNA (DANCR) is a newly identified lncRNA dysregulated and presenting multiple oncogenic activities in many human cancers (Thin *et al.*, 2018). The functions and mechanisms of DANCR in breast cancer, however, have only been minimally explored. One study by Sha *et al. *(2017) showed that DANCR was up‐regulated in TNBC tissues and cell lines, associated with worse TNM stages and overall survival, essentially contributing tumor cell growth and metastasis, and by regulating the promoter binding of enhancer of zeste homolog 2 (EZH2), stimulating the expressions of CSC markers, ATP Binding Cassette Subfamily G Member 2 (ABCG2), and aldehyde dehydrogenase 1 (ALDH1). Although Sha *et al.*’s study supports the oncogenic nature of DANCR in breast cancer, it is not known whether DANCR essentially regulates the crosstalk among EMT, cancer stemness, and inflammation, and if so, what mechanisms are involved.

In the present study, we specifically examined the functional roles of DANCR in regulating EMT, cancer stemness, and inflammation, and explored the molecular mechanisms mediating the actions of DANCR. We report for the first time that DANCR essentially contributed to all three malignant phenotypes of breast cancer, which, on the molecular level, was mediated through EZH2‐controlled epigenetic regulation of suppressor of cytokine signaling 3 (SOCS3) *via* H3K27 trimethylation. Therefore, inhibiting DANCR or up‐regulating SOCS3 may simultaneously target all three metastasis‐related phenotypes and improve the prognosis of breast cancer patients.

## 2 Materials and methods

### 2.1 Human tissues and cell lines

The protocols for the use of human tissues in this study were approved by the Ethics Committee of Central South University (Hunan, China) and were conducted in accordance with the Declaration of Helsinki. Written consent was obtained from all participants. A total of 46 women diagnosed with breast cancer and admitted into our hospital between 2016 and 2018 were recruited into this study. A cohort of 46 pairs of breast cancer tissues and matching para‐tumor normal tissues was acquired during surgery and confirmed by pathological examinations. All tissues were snap‐frozen in liquid nitrogen till further use.

The normal human breast epithelial cell line MCF10A, and breast cancer cell lines MCF7, T47D, MDA‐MB‐231, and MDA‐MB‐468, were purchased from the Cell Bank of Type Culture Collection (Chinese Academy of Sciences, Shanghai, China). MCF10A cells were cultured in Dulbecco’s modified Eagle’s medium/F12 (DMEM/F12; Invitrogen, Carlsbad, CA, USA) containing 5% horse serum (Invitrogen), 20 ng·mL^−1^ recombinant human EGF (PeproTech, Rocky Hill, NJ, USA), 0.5 mg·mL^−1^ hydrocortisone (Sigma, St. Louis, MO, USA), 100 ng·mL^−1^ cholera toxin (Sigma), 10 µg·mL^−1^ insulin (Sigma), and 1% penicillin/streptomycin (Invitrogen). The four breast cancer cell lines were cultured in DMEM (Invitrogen) containing 10% FBS (Invitrogen) and 1% penicillin/streptomycin. All cells were maintained in a sterile humidified atmosphere containing 5% CO_2_ at 37 °C.

### 2.2 Reverse transcription followed by qRT‐PCR

TRIzol reagent (Invitrogen) was used to extract total RNA from frozen tissues or cultured cells. The Takara reverse transcription system (Dalian, China) was used to synthesize cDNA from total RNA. Quantitative real‐time PCR (qRT‐PCR) was performed using iQTM SYBR® Green Supermix (Bio‐Rad, Hercules, CA, USA) on an ABI‐7500 thermocycler. Primer sequences used for qRT‐PCR analysis are listed in Table 1. The relative expression of a target gene to that of the internal control was calculated following the 2^−ΔΔCt^ method (Livak and Schmittgen, 2001).

**Table 1:** Primer sequences used for qRT‐PCR analysis.

Gene	Forward primer (5′–3′)	Reverse primer (5′–3′)
DANCR	GCGCCACTATGTAGCGGGTT	TCAATGGCTTGTGCCTGTAGTT
CD44	TTACAGCCTCAGCAGAGCAC	TGACCTAAGACGGAGGGAGG
CD133	CAGAGTACAACGCCAAACCA	AAATCACGATGAGGGTCAGC
OCT3/4	ATGTGGTCCGAGTGTGGTTC	ACAGTGCAGTGAAGTGAGGG
NANOG	CTCCAACATCCTGAACCTCAGC	CGTCACACCATTGCTATTCTTCG
IL‐6	AGACAGCCACTCACCTCTTCAG	TTCTGCCAGTGCCTCTTTGCTG
TGF‐β	TACCTGAACCCGTGTTGCTCTC	GTTGCTGAGGTATCGCCAGGAA

### 2.3 Construction of lentivirus and generation of stable cells

Differentiation antagonizing non‐protein coding RNA shRNA and negative control shRNA were synthesized by GenePharma (Shanghai, China). For virus construction, shRNA were subcloned into pLKO.1 vector. The target sequences of shRNA are listed in Table 2, and shRNA#3 was used for all follow‐up experiments. For virus packaging, the pLKO.1‐shDANCR plasmid was then transfected into HEK293T cells with psPAX2 packaging plasmid and pMD2.G envelope plasmid using Lipofectamine 3000 (Invitrogen). At 48 h after the transfection, the supernatant containing lentivirus was harvested from the culture and centrifuged at 500 ***g*** for 5 min to remove any cell debris. For lentiviral infection, target cells were incubated with lentivirus in the presence of polybrene (8 µg·mL^−1^; Sigma) overnight. Then, the cells were cultured in fresh complete growth medium for 48 h and selected.

**Table 2:** Target sequence of DANCR shRNA.

ShRNA No.	Target sequence
shRNA#1	GCTGGTAAAGAAATGGATTAG
shRNA#2	GCCCTGAATACACACCCAAGC
shRNA#3	GGATGACCGCTTTGCACATCA
shRNA#4	GCTCTTGATATGTCATCACCG

To overexpress SOCS3, the human SOCS3 cDNA was cloned into pcDNA3.1 vector and transiently transfected into target cells using Lipofectamine 3000 (Invitrogen) following the manufacturer’s instructions.

### 2.4 Cell viability assay

The MTT assay was performed to measure cell viability. Briefly, 2 × 10^3^ cells growing in log phase were seeded into 96‐well plates and incubated for 6, 12, 24, 48, or 72 h, respectively. MTT solution (20 µL·well^−1^, 5 mg·mL^−1^ in PBS; Sigma) was added to each well, and cells were incubated at 37 °C for a further 3 h. Cells were then incubated with 100 µL of DMSO (Sigma) in the dark for 2 h. Finally, the A_490_ from each well (proportional to the number of live cells) was measured with a Microplate Reader Bio‐Rad 550.

### 2.5 Cell‐cycle analysis with propidium iodide (PI)

Cells were collected, washed with PBS three times, and fixed in 70% ethanol overnight. For cell‐cycle analysis, fixed cells were stained using FxCycle™ PI/RNase Staining Solution (Thermo Fisher Scientific, Waltham, MA, USA) according to the manufacturer’s instructions and analyzed using a FACSCalibur flow cytometer (BD Biosciences, San Jose, CA, USA).

### 2.6 Transwell migration/invasion assay

Transwell inserts (pore size: 8.0 µm; Corning, Lowell, MA, USA) coated with or without Matrigel (BD Biosciences) were used to measure the migration and invasion of cells, respectively. Briefly, 1 × 10^5^ target cells in serum‐free DMEM medium were seeded into the top well and 500 μL of DMEM containing 10% FBS was added to the lower chamber. After 24 h, the non‐migrating or non‐invading cells from the upper side of the membrane were cleared using cotton swabs, and the invaded cells were fixed in 95% methanol at room temperature for 10 min and stained with 1% crystal violet for 5 min. Images of migrating or invading cells were taken under an inverted microscope (×100), with the number counted and averaged from at least five random fields.

### 2.7 *In vivo* mouse models

Protocols for animal experiments were approved by the Institutional Animal Care and Use Committee of Central South University, China. Male Balb/c nu/nu mice (4–5 weeks old, 14–16 g) were purchased from SLAC Laboratory Animal Co. Ltd (Hunan, China) and housed in a specific‐pathogen‐free facility. To establish the xenograft model, target cells were subcutaneously injected into the dorsal flank region of each mouse on Day 0 (1 × 10^6^ cells per injection in 100 µL of saline). From Day 15, we measured the length (*L*) and width (*W*) of each xenograft tumor using a Vernier caliper every 4 days, and calculated the tumor volume (*V*) as *V* = 1/2 × *L *× *W* ^2^. All mice were sacrificed on day 35 after the initial inoculation of cells. To study lung metastasis, target cells were intravenously injected into the tail vein of each mouse on Day 0 (2 × 10^5^ cells per injection in 100 µL of saline). On day 35, all mice were sacrificed, with lungs isolated, fixed in 10% formalin, and counted for surface metastatic nodules under a dissecting microscope.

### 2.8 Mammosphere formation assay

The mammosphere formation assay was performed as described previously (Lombardo *et al.*, 2015). Briefly, single‐cell suspensions of target cells were seeded into six‐well ultra‐low attachment plates (Corning) and cultured in mammosphere media [DMEM/F12 supplemented with 2 mm l‐glutamine, 100 U·mL^−1^ penicillin, 100 U·mL^−1^ streptomycin, 20 ng·mL^−1^ recombinant human EGF, 10 ng·mL^−1^ recombinant human basic fibroblast growth factor (PeproTech) and 1 × B27 supplement (Thermo Fisher Scientific)] for 10 days. The number of spheroids with a diameter of > 40 µm was counted under an inverted microscope (×40). To examine the self‐renewal capability of the cells, the first‐generation spheroids were collected, dissociated, and re‐plated under the same conditions for three more generations.

### 2.9 Western immunoblots

RIPA buffer (Thermo Fisher Scientific) was used to extract total proteins from cells. Upon electrophoresis on 10% SDS/PAGE gel and transferral to a polyvinylidene difluoride membrane, target proteins were detected using the following primary antibodies (all from Cell Signaling Technology, Danvers, MA, USA, unless otherwise indicated) against Snail1 (1 : 1000; #3879), Slug (1 : 1000; #9585), MMP2 (1 : 1000; #409994), MMP9 (1 : 1000; #13667), E‐cadherin (1 : 1000; #14472), Vimentin (1 : 1000; #5741), CD44 (1 : 1000; #37259), CD133 (1 : 1000; #64326), OCT3/4 (1 : 1000; sc‐5279; Santa Cruz Biotechnology, Santa Cruz, CA, USA), NANOG (1 : 1000; #8822), p‐p65 (1 : 1000; #3033), p65 (1 : 1000; #8242), Phospho STAT3 (1 : 1000; #9145), and STAT3 (1 : 1000; #9139), SOCS3 (1 : 1000; #2923), Ezh2 (1 : 1000; #5246), H3K27me3 (1 : 1000; #9733), H3 (1 : 1000; #4499) or GAPDH (1 : 5000; #5174; internal control) at 4 °C overnight. After incubation with horseradish peroxidase‐conjugated secondary antibodies, the signal was detected using the enhanced chemiluminescence system (Beyotime, Jiangsu, China).

### 2.10 ChIP assay

ChIP assay was performed using SimpleChIP Kit (Cell Signaling Technology) according to the protocols provided by the manufacturer. Briefly, cells were crosslinked with 1% formaldehyde and lysed to prepare nuclei. Chromatin was then partially digested in micrococcal nuclease followed by sonication to generate DNA/protein fragments of 150–900 base pairs (bp) in length. Upon incubating the digested chromatin with anti‐EZH2 antibody (#5246), anti‐H3K27me3 antibody (#9733) or normal rabbit IgG (negative control; all from Cell Signaling Technology) at 4 °C overnight, the immune complexes were pulled down using ChIP‐grade protein G magnetic beads. After eluting chromatin from the antibody/protein G magnetic beads, DNA was purified using the spin column provided with the kit and examined using PCR with the following primers: SOCS3 forward CGCTTCGGGACTAGGTAGGA, and SOCS3 reverse AGAAACCGGGAAAAGCTCCC.

### 2.11 RNA immunoprecipitation (RIP assay)

The interaction between DANCR and EZH2 was measured using EZ‐Magna RNA immunoprecipitation (RIP assay) RNA‐Binding Protein Immunoprecipitation Kit (Sigma) following the manufacturer’s protocols. Antibody for RIP assays of EZH2 (#5246) was from Cell Signaling Technology.

### 2.12 Statistical analysis

All data were analyzed using graphpad prism 6.0 (Graphpad, San Diego, CA, USA) and presented as the mean ± SD of at least three independent experiments (for *in vitro* assays) or multiple mice within each group (for *in vivo* xenograft model). Differences between experimental groups were assessed by Student’s *t*‐test or one‐way ANOVA. A *P*‐value of < 0.05 was considered statistically significant.

## 3 Results

### 3.1 DANCR was up‐regulated in breast cancer tissues and cell lines, specifically in cancers of advanced pathological grades or associated with lymph node metastasis

**Figure 1:**
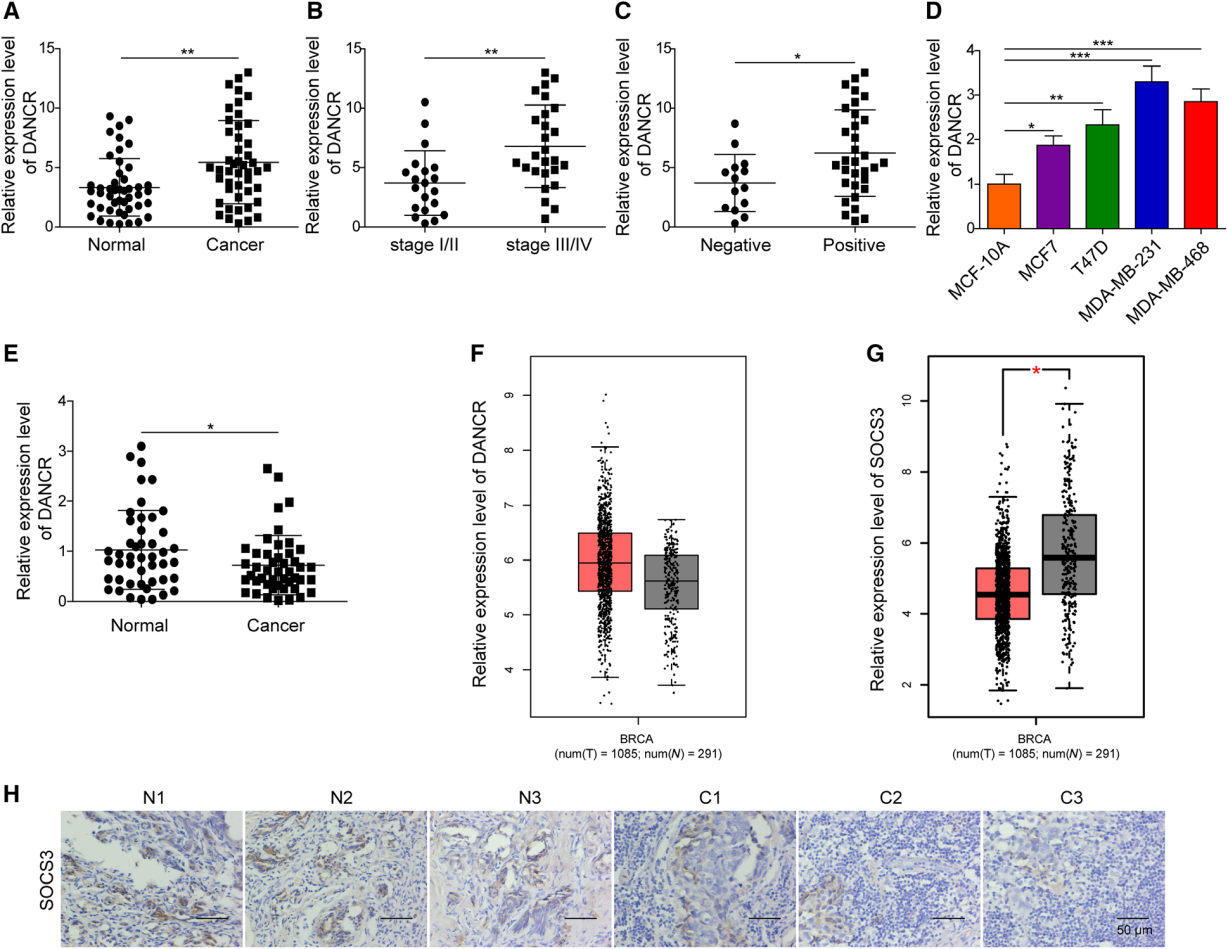
DANCR was up‐regulated in breast cancer tissues or cells. The expression of DANCR was examined by qRT‐PCR and is presented as a relative ratio to that of U6 (internal control). The expression of DANCR was compared between 46 pairs of breast cancer tissues and the matching para‐tumor normal tissues (A), between breast cancer tissues of low (stage I/II, *n* = 20) and high (stage III/IV, *n* = 26) pathological grades (B), between breast cancer tissues showing negative (*n* = 14) and positive (*n* = 32) lymph node metastasis (C), and between indicated breast epithelial cell lines (D). (E) The expression of SOCS3 was examined by qRT‐PCR and compared between 46 pairs of breast cancer tissues and matching normal tissues. (F,G) Analysis of the TCGA database showed that DANCR was up‐regulated, whereas SOCS3 was down‐regulated in breast cancer. (H) The expression of SOCS3 was examined by immunohistochemistry in breast cancer vs. matching normal tissues. Representative images from three pairs of matching tissues (N for normal and C for cancer) are shown. *n* = 3, data are shown as mean ± SD. Student’s *t‐*test was used to determine statistical significance: *P < 0.05, **P < 0.01, ***P < 0.001.

We collected 46 pairs of human breast cancer tissues and the matching adjacent normal tissues to assess the expression of DANCR in breast cancer. qRT‐PCR analysis showed that DANCR was significantly up‐regulated in breast cancer tissues compared with normal tissues (*P* = 0.009; Fig. 1A). Further classification of breast cancer tissues according to the TNM stages revealed that the expression of DANCR was markedly elevated in advanced tumors (*P* < 0.05, comparing tumors of stage III/IV (*n* = 26) with those of stage I/II (*n* = 20); Fig. 1B) and also in those positive for lymph node metastasis (*n* = 32; *P* < 0.05, when compared with tumors negative for lymph node metastasis (*n* = 14); Fig. 1C). Consistently, when examining DANCR expression in breast epithelial cells of different malignancy, we found that DANCR was minimally expressed in normal breast epithelial cells MCF10A, increased in breast cancer cells of low malignancy, MCF7 and T47D (*P* < 0.05, when compared with MCF10A cells), and was further up‐regulated in highly malignant TNBC cells, MDA‐MB‐231 and MDA‐MB‐468 (*P* < 0.05, when comparing MDA‐MB‐231 with MCF10A, MCF7 or T47D cells, and comparing MDA‐MB‐468 with MCF10A or MCF7 cells; *P *> 0.05, when comparing T47D with MDA‐MB‐468 cells; Fig. 1D). These findings support the pro‐malignant and pro‐metastatic activities of DANCR in breast cancer.

Further analysis of The Cancer Genome Atlas (TCGA, https://www.cancer.gov/tcga) database showed consistent results; DANCR was up‐regulated in breast cancer tissues, whereas SOCS3 was down‐regulated (Fig. 1F,G). IHC representative images showed that SOCS3 is down‐regulated in cancer tissue compared with normal tissue (Fig. 1H).

### 3.2 In malignant breast cancer cells, DANCR essentially controlled cell viability and migration/invasion *in vitro*, as well as xenograft growth *in vivo*

**Figure 2:**
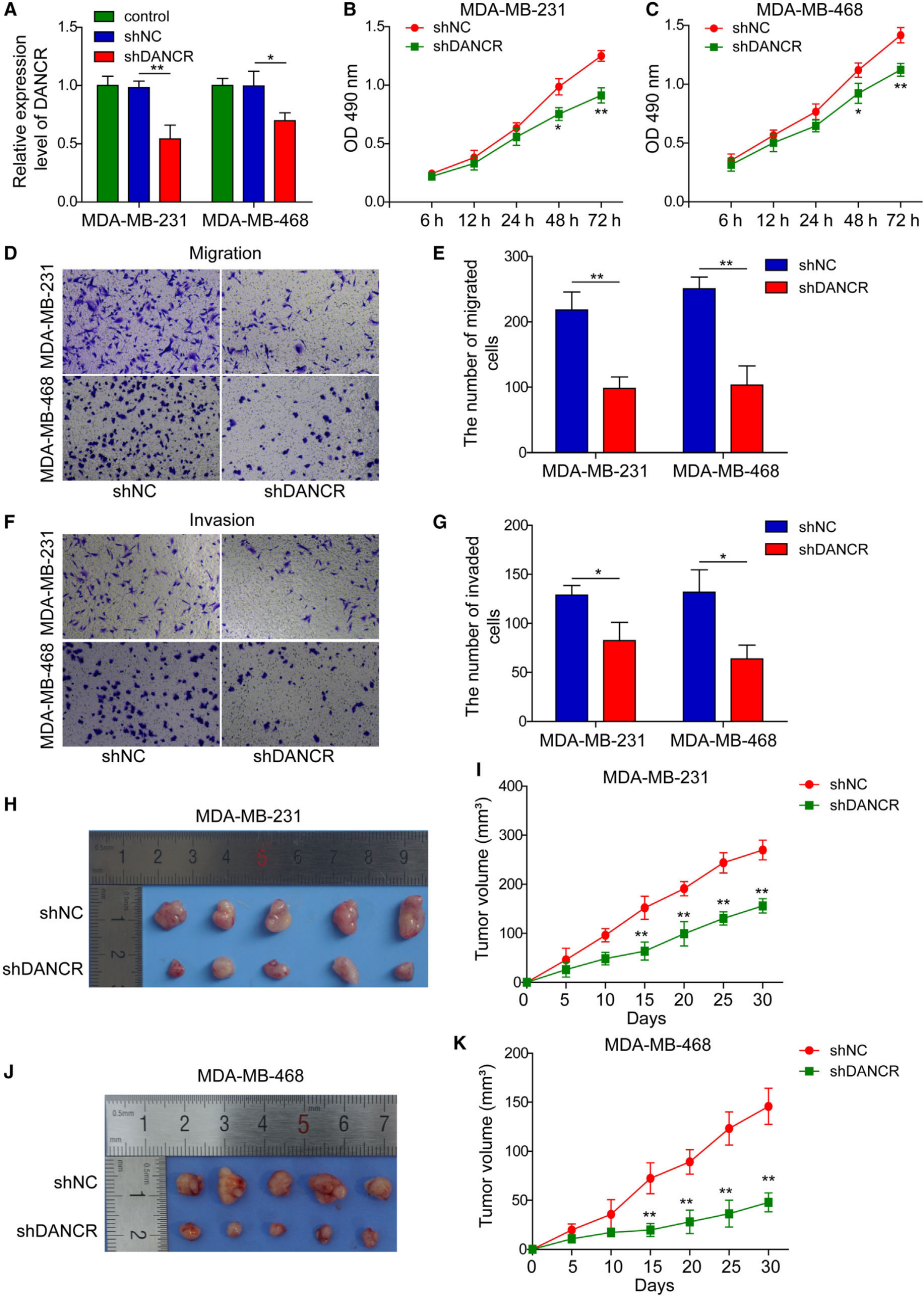
DANCR was essential for maintaining *in vitro* viability, migration, and invasion, and *in vivo* xenograft growth of malignant breast cancer cells. shDANCR was stably transfected into MDA‐MB‐231 and MDA‐MB‐468 cells; non‐transfected (control) or shNC‐transfected cells were examined in parallel. (A) qRT‐PCR shows reduction of DANCR in shDANCR cells. (B,C) MTT assay showed shDANCR significantly reduced the viability of indicated breast cancer cells. Transwell migration (D,E) and invasion (F,G) assay showed shDANCR potently inhibited the migration and invasion of breast cancer cells. Representative images of migrated (D) or invaded (F) cells are shown on the left and the quantification on the right (E,G). (H–K) Xenograft tumors (*n* = 5/group) were generated from shNC or shDANCR cells. (H,J) Photographs for xenografts from indicated groups. (I,K) The growth curve of xenografts from indicated groups. *n* = 3, data are shown as mean ± SD. Student’s *t‐t*est was used to determine statistical significance: *P < 0.05, **P < 0.01.

To understand the functional significance and explore the underlying mechanisms of DANCR in breast cancer, we adopted both loss‐of‐function and gain‐of‐function strategies. For loss‐of‐function strategy, we stably reduced the endogenous DANCR level in highly malignant MDA‐MB‐231 and MDA‐MB‐468 cells using shRNA‐mediated gene silencing. Figure 2A showed that shDANCR lowered the expression of DANCR in these cells by ~ 30–50% (*P* < 0.05, when compared with the parental control or shNC‐expressing cells. Although the knockdown of DANCR by shDANCR was not ideal, by comparing several malignant phenotypes between shDANCR and shNC cells, we found that knocking down DANCR significantly reduced the viability (Fig. 2B,C), induced cell‐cycle arrest at G0/G1 phase (Fig. S1), and inhibited both the migration (Fig. 2D,E) and the invasion (Fig. 2F,G) of both MDA‐MB‐231 and MDA‐MB‐468 cells, suggesting that DANCR essentially maintained multiple malignant behaviors of breast cancer cells.

In addition to examining the effects of DANCR on malignant breast cancer cells cultured *in vitro*, we established xenograft models using shDANCR or shNC malignant breast cancer cells (MDA‐MB‐231 and MDA‐MB‐468) and monitored the growth of xenograft tumors *in vivo.* Consistent with the growth‐inhibitory effect of shDANCR *in vitro*, xenografts derived from shDANCR (MDA‐MB‐231 and MDA‐MB‐468) cells displayed significantly slower growth than those derived from shNC cells (*P* < 0.05; Fig. 2H–K). To assess the effects of DANCR on cancer metastasis *in vivo,* we injected shDANCR or shNC malignant breast cancer cells (MDA‐MB‐231 and MDA‐MB‐468) through the tail vein and found that knocking down DANCR in malignant breast cancer cells significantly reduced the number of metastatic nodules formed in lung (Fig. S2A,B). Taken together, these data support oncogenic and pro‐metastatic activities of DANCR *in vivo*.

### 3.3 DANCR critically regulated EMT and cancer stemness of malignant breast cancer cells

**Figure 3:**
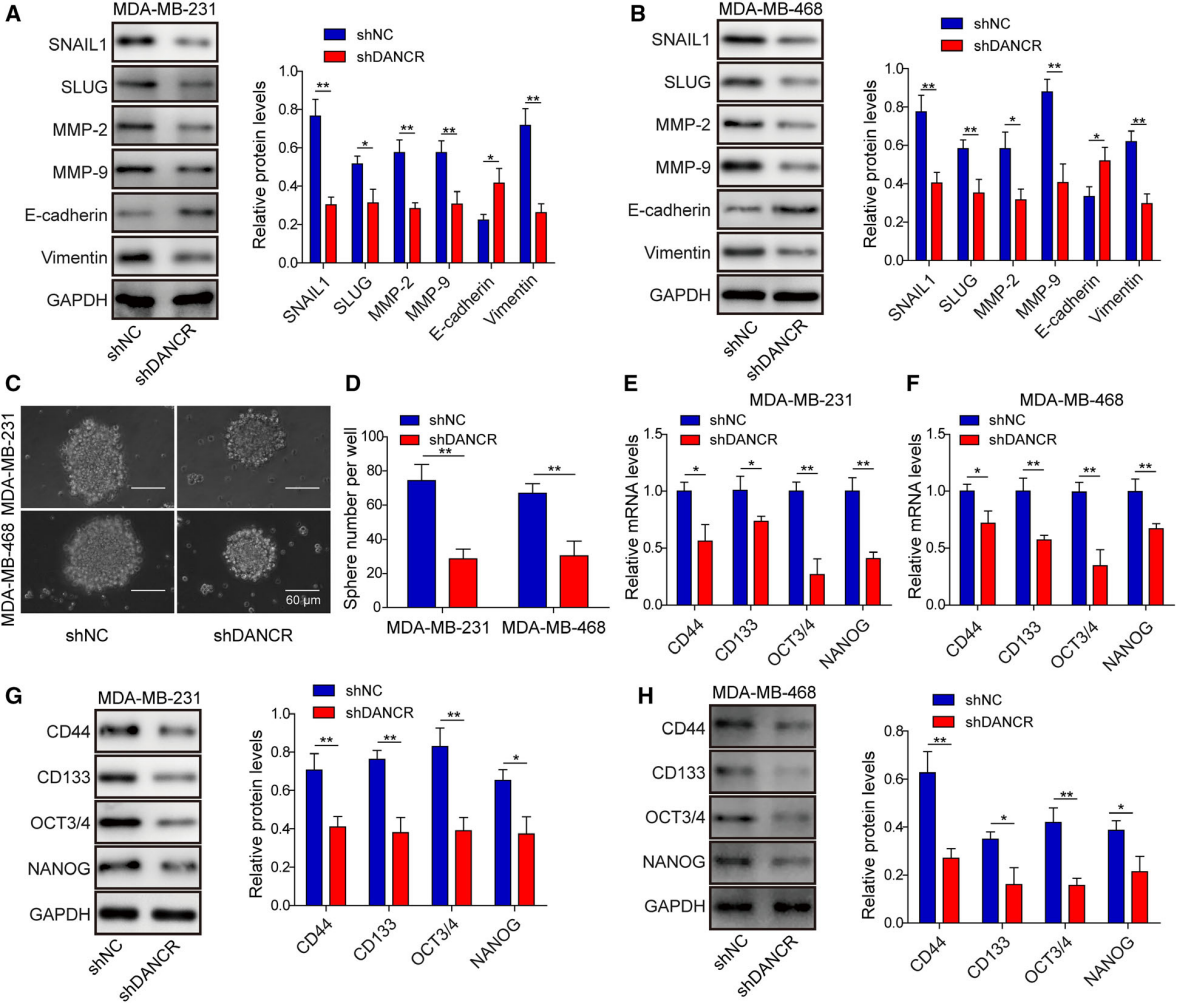
DANCR essentially regulated EMT and cancer stemness of malignant breast cancer cells. (A,B) Western blot on EMT‐related biomarkers SNAIL1, SLUG, MMP‐2, MMP‐9, E‐cadherin, and Vimentin showed that shDANCR inhibited EMT in MDA‐MB‐231 and MDA‐MB‐468 cells. Representative western images are shown on the left and the quantification on the right. (C,D) Mammosphere assay showed that shDANCR significantly inhibited mammosphere formation. Representative images of mammosphere formed in each group are shown in (C) and the quantification on the number of mammospheres in (D). (E–H) qRT‐PCR (E,F) and western blot (G,H) show shDANCR inhibited the expression of stemness‐related biomarkers CD44, CD133, OCT3/4, and NANOG, on mRNA and protein level in indicated breast cancer cells. *n* = 3, data are shown as mean ± SD. Student’s *t‐t*est was used to determine statistical significance: *P < 0.05, **P < 0.01.

Epithelial‐mesenchymal transition is closely associated with cancer stemness, and both phenotypes contribute significantly to tumor metastasis, recurrence, and drug resistance (Wang *et al.*, 2015). To assess whether DANCR regulates EMT or cancer stemness of malignant breast cancer cells, we first examined the expressions of EMT‐related biomarkers, including SNAIL1, SLUG, MMP‐2, MMP‐9, E‐cadherin, and Vimentin in shDANCR vs. shNC (MDA‐MB‐231 and MDA‐MB‐468) cells. As shown in Fig. 3A,B, shDANCR markedly reduced the levels of mesenchymal biomarkers SNAIL1, SLUG, MMP‐2, MMP‐9, and Vimentin, but elevated the level of the epithelial biomarker E‐cadherin (*P* < 0.05, when compared with shNC cells). To investigate the impact of DANCR on CSC activity, we performed the mammosphere formation assay and found that shDANCR significantly reduced the size (Fig. 3C) and number (Fig. 3D) of mammospheres formed (*P* < 0.05, when compared with shNC cells). The inhibition of targeting DANCR on self‐renewal of CSCs was not limited to the first generation of mammospheres, but persisted till at least the 4th generation (Fig. S3A,B). Consistently, the expressions of CSC‐related biomarkers CD44, CD133, OCT3/4, and NANOG were all remarkably reduced on both mRNA and protein levels in shDANCR cells (*P* < 0.05, when compared with shNC cells; Fig. 3E–H). These data suggest that DANCR critically regulates both EMT and stemness of malignant breast cancer cells.

### 3.4 Knocking down DANCR in malignant breast cancer cells reduced the production of inflammatory cytokines, blocked the binding of EZH2 to SOCS3 promoter, and up‐regulated SOCS3

**Figure 4:**
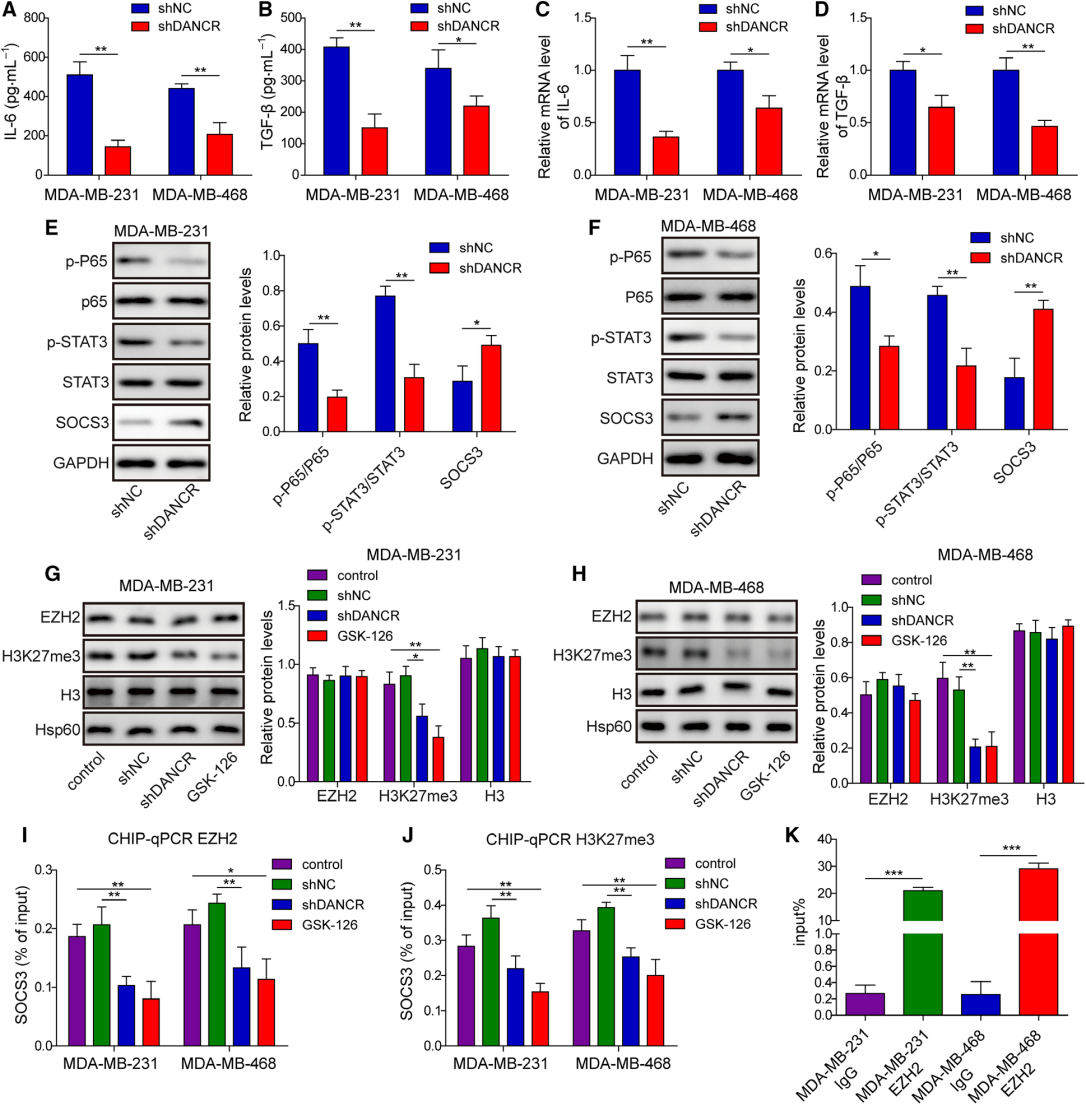
DANCR critically controlled inflammation and targeted SOCS3 expression through EZH2‐mediated epigenetic regulation. ELISA assay showed that shDANCR potently reduced the secretions of IL‐6 (A) and TGF‐β (B) into the conditioned medium of indicated breast cancer cells. qRT‐PCR revealed reduced expressions of IL‐6 (C) and TGF‐β (D) in shDANCR cells. (E,F) Western blot showed that shDANCR markedly inhibited the activations of p65 and STAT3, but increased the expression of SOCS3 in malignant breast cancer cells. (G,H) Western blot on EZH2, H3K27me3, H3, and Hsp60 revealed specific reductions of H3K27me3 in shDANCR or GSK‐126‐treated cells. ChIP assay showed that the binding of EZH2 (I) and H3K27me3 (J) to the promoter of SOCS3 was significantly reduced in shDANCR or GSK‐126‐treated cells. (K) RNA immunoprecipitation assay revealed the specific interaction between DANCR and EZH2 in malignant breast cancer cells. IgG was used as the negative control. *n* = 3, data are shown as mean ± SD. Student’s *t*‐test (A–F) and one‐way ANOVA (G–K) were used to determine statistical significance: *P < 0.05, **P < 0.01, ***P < 0.001.

Several signaling pathways, including IL‐6/JAK2/STAT3 and TGF‐β/Smad (Katsuno *et al.*, 2013), as well as the crosstalk between them (Liu *et al.*, 2014), contributed to EMT and cancer stemness. As knocking down DANCR inhibited EMT and stemness of malignant breast cancer cells, we examined the effect of shDANCR on the production of IL‐6 and TGF‐β from these cells. First, the secretion of IL‐6 and TGF‐β into the conditioned medium of shDANCR (both MDA‐MB‐231 and MDA‐MB‐468) cells was significantly lower than secretion into the conditioned medium of shNC cells (*P* < 0.05; Fig. 4A,B). Consistently, shDANCR significantly reduced the steady‐state mRNA levels of both IL‐6 and TGF‐β in MDA‐MB‐231 and MDA‐MB‐468 cells (*P* < 0.05, when compared with the corresponding shNC cells; Fig. 4C,D).

Next, we measured the expressions of several inflammation‐related signaling molecules in MDA‐MB‐231 and MDA‐MB‐468 cells (Fig. 4E,F). We found that the activation of NF‐κB (as represented by p‐p65 level) and STAT3 (as represented by p‐STAT3 level) were strongly reduced, whereas the expression of SOCS3 was significantly increased in shDANCR cells compared with shNC cells, with a more prominent increase observed in MDA‐MB‐468 than in MDA‐MB‐231 cells (*P* < 0.05). To understand the molecular mechanisms elevating SOCS3 expression, we focused on EZH2, a lysine methyltransferase that synergizes with DANCR to silence target genes epigenetically through methylation of H3K27me3 (Jia *et al.*, 2016). When examining shDANCR vs. shNC (both MDA‐MB‐231 and MDA‐MB‐468) cells, we found that although the total level of EZH2 was not altered, the level of H3K27me3 was potently reduced in shDANCR cells to a level comparable to that achieved by the EZH2 inhibitor GSK‐126 (*P* < 0.05; Fig. 4G,H). Consistently, ChIP‐qPCR analysis showed that in both shDANCR‐ and GSK‐126‐treated MDA‐MB‐231 and MDA‐MB‐468 cells, the binding of EZH2 or H3K27me3 to the promoter of SOCS3 was markedly reduced (*P* < 0.05, when compared with the control or shNC cells; Fig. 4I,J), although the overall binding between SOCS3 and EZH2 or H3K27me3 was relatively low, as indicated by the percentage of input. Furthermore, RIP assay showed that in both MDA‐MB‐231 and MDA‐MB‐468 cells, DANCR bonded to EZH2 (Fig. 4K). These data suggest that by binding with EZH2, DANCR participated in EHZ2‐mediated epigenetic repression of SOCS3 in malignant breast cancer cells.

### 3.5 Up‐regulating DANCR in normal breast epithelial cells or breast cancer cells of low malignancy stimulated the viability and migration/invasion *in vitro*, and xenograft growth *in vivo*

**Figure 5:**
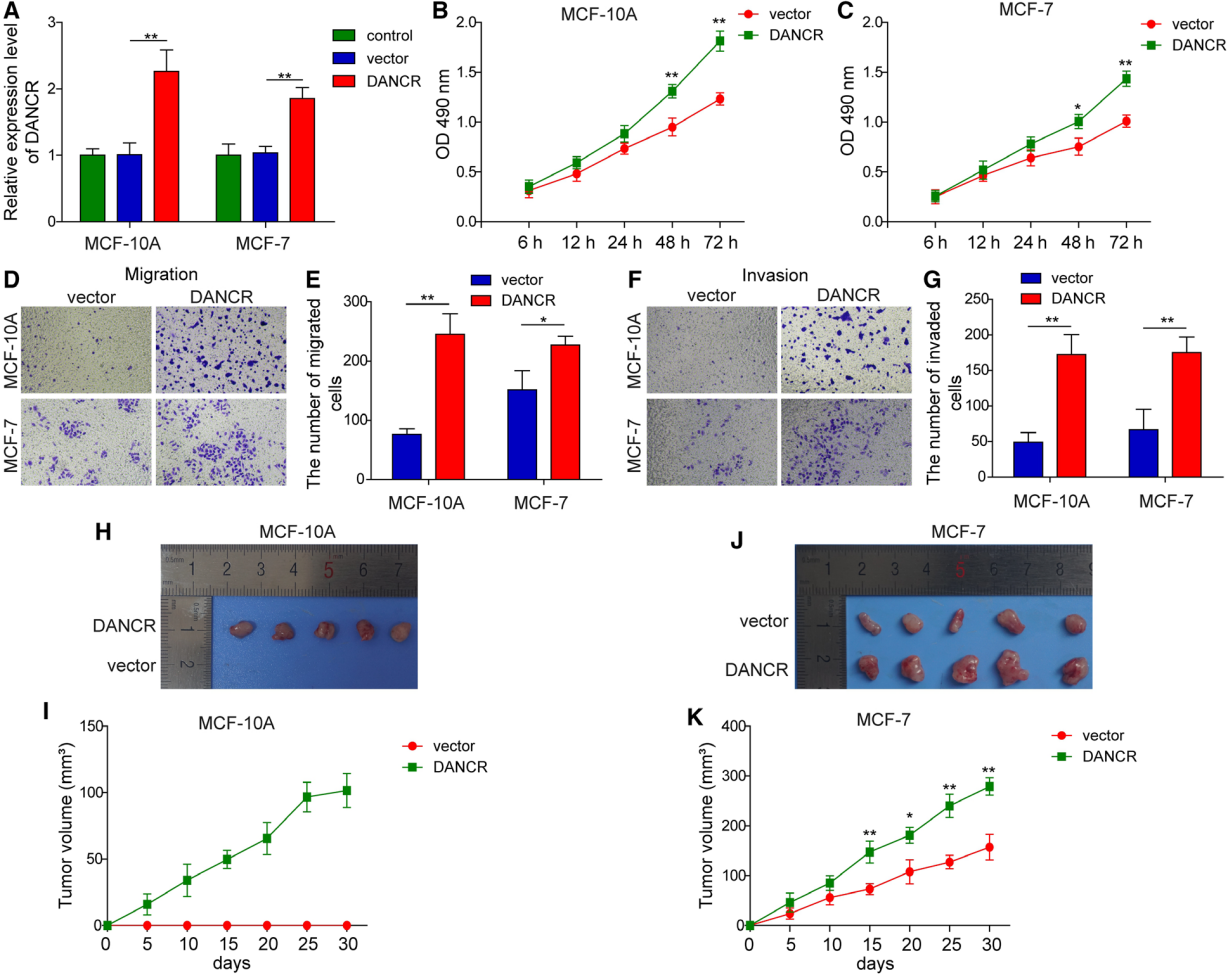
DANCR was sufficient to promote *in vitro* viability, migration, and invasion, and *in vivo* xenograft growth of normal breast epithelial cells or breast cancer cells of low malignancy. DANCR was overexpressed in MCF10A and MCF‐7 cells; parental (control) or vector‐transfected cells (vector) were examined in parallel. (A) RT‐qPCR showed elevation of DANCR level in shDANCR cells. (B,C) MTT assay showed that DANCR significantly boosted the viability of indicated cells. Transwell migration (D,E) and invasion (F,G) assay showed that DANCR stimulated the migration and invasion of indicated cells. Representative images of migrated (D) or invaded (F) cells are shown on the left and the quantification on the right (E,G). (H,K) Xenograft tumors (*n* = 5/group) were generated from vector or DANCR cells. (H,J) Photographs for xenografts from indicated groups. (I,K) The growth curve of xenografts from indicated groups. *n* = 3, data were shown as mean ± SD. Student’s *t*‐test was used to determine statistical significance: *P < 0.05, **P < 0.01.

In addition to knocking down DANCR in malignant breast cancer cells, we also stably increased its level in normal breast epithelial cells, MCF10A, or breast cancer cells of low malignancy, MCF7, using lentivirus‐mediated gene expression. When compared with parental (control) cells or cells infected with lentivirus from empty vector (vector), DANCR‐expressing (DANCR) cells presented an approximately twofold higher expression of DANCR (*P* < 0.05; Fig. 5A). Corresponding to the increase in DANCR in these cells, *in vitro* viability (Fig. 5B,C), migration (Fig. 5D,E), and invasion (Fig. 5F,G) were significantly stimulated (*P < *0.05, when compared with vector cells), as was the xenograft growth (Fig. 5H–K) and lung metastasis (Fig. S2C,D) *in vivo* (*P < *0.05, when compared with vector cells), suggesting that DANCR was sufficient to induce malignant transformation of normal breast epithelial cells and boost the malignancy of breast cancer cell lines of low malignancy.

### 3.6 DANCR was sufficient to promote EMT and cancer stemness in normal breast epithelial cells or breast cancer cells of low malignancy

**Figure 6:**
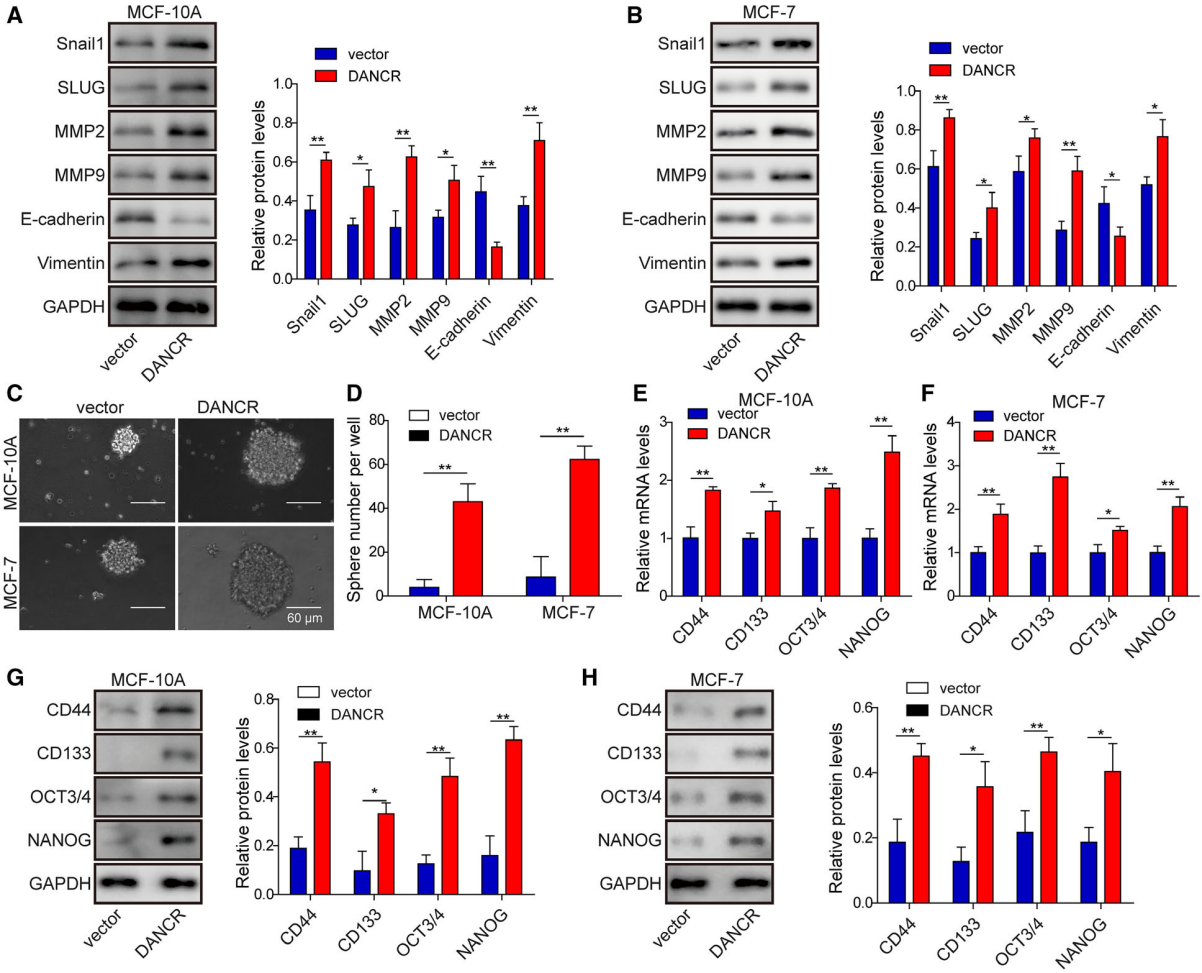
DANCR stimulated EMT and cancer stemness of normal breast epithelial cells or breast cancer cells of low malignancy. (A,B) Western blot on EMT‐related biomarkers SNAIL1, SLUG, MMP‐2, MMP‐9, E‐cadherin, and Vimentin showed that DANCR stimulated EMT in MCF‐10A and MCF‐7 cells. Representative western images are shown on the left and the quantification on the right. (C,D) Mammosphere assay showed that DANCR significantly promoted mammosphere formation. Representative images of mammosphere formed in each group are shown in (C) and the quantification on the number of mammospheres in (D). (E–H) qRT‐PCR (E,F) and western blot (G,H) showed DANCR increased expressions of stemness‐related biomarkers CD44, CD133, OCT3/4, and NANOG, on mRNA level and protein level in indicated cells. *n* = 3, data are shown as mean ± SD. Student’s *t*‐test was used to determine statistical significance: *P < 0.05, **P < 0.01.

When it comes to EMT, we found that DANCR overexpression up‐regulated the expression of mesenchymal biomarkers SNAIL1, SLUG, MMP‐2, MMP‐9, and Vimentin, while down‐regulating the expression of E‐cadherin (*P* < 0.05, when compared with vector cells; Fig. 6A,B). Furthermore, DANCR‐expressing cells generated larger (Fig. 6C) and more mammospheres (Fig. 6D), which corresponded to the elevations of CSC biomarkersg CD44, CD133, OCT3/4, and NANOG (on both mRNA and protein levels; *P* < 0.05, when compared with vector cells; Fig. 6E–H), suggesting the potency of DANCR in promoting EMT and cancer stemness in normal breast epithelial cells or breast cancer cells of low malignancy. The induction of overexpressing DANCR on self‐renewal of CSC was not limited to the first generation of mammospheres, but persisted till at least the third generation, although the ability decayed. For the fourth generation of mammospheres, we detected no significant difference in MCF10A cells, suggesting that DANCR induced generation of mammospheres lost in subculture.

### 3.7 Elevating DANCR in normal breast epithelial cells or breast cancer cells of low malignancy increased the production of inflammatory cytokines, stimulated the binding of EZH2 to SOCS3 promoter, and down‐regulated SOCS3

**Figure 7:**
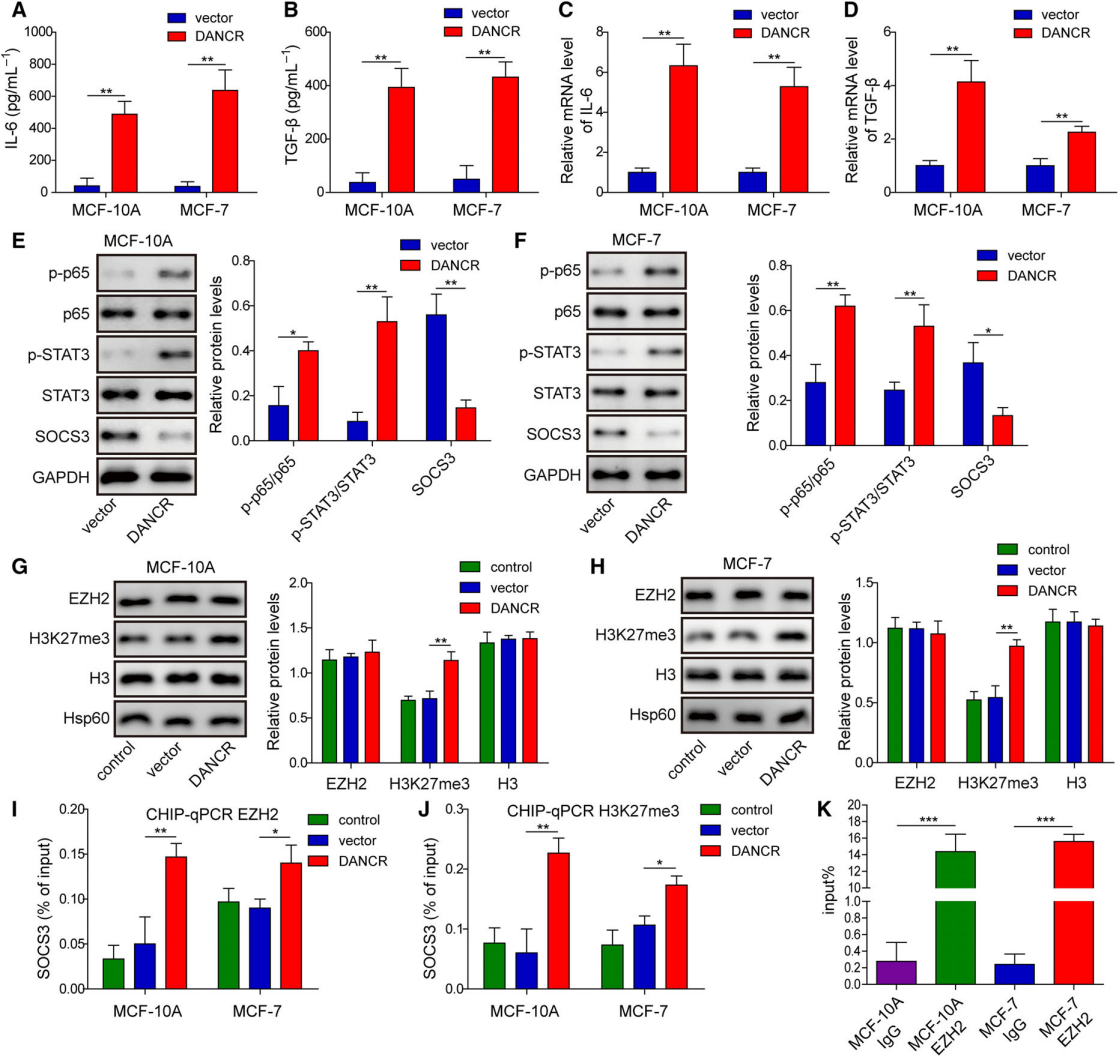
DANCR sufficiently stimulated inflammation and targeted SOCS3 expression through EZH2‐mediated epigenetic regulation in normal breast epithelial cells or breast cancer cells of low malignancy. ELISA assay showed that DANCR overexpression potently stimulated the secretions of IL‐6 (A) and TGF‐β (B) into the conditioned medium of indicated cells. RT‐qPCR revealed increased expressions of IL‐6 (C) and TGF‐β (D) in DANCR cells. (E,F) Western blot showed that DANCR markedly promoted the activation of p65 and STAT3, but reduced the expression of SOCS3 in indicated cells. (G,H) Western blot on EZH2, H3K27me3, H3, and Hsp60 revealed specific increases of H3K27me3 in DANCR cells. ChIP assay showed that the binding of EZH2 (I) and H3K27me3 (J) to the promoter of SOCS3 was significantly increased in DANCR cells. (K) RNA immunoprecipitation assay revealed the specific interaction between DANCR and EZH2 in MCF‐10A and MCF‐7. IgG was used as the negative control. *n* = 3, data are shown as mean ± SD. Student’s *t*‐test (A–F) and one‐way ANOVA (G–K) were used to determine statistical significance: *P < 0.05, **P < 0.01, ***P < 0.001.

To assess the significance and mechanisms of DANCR in regulating inflammation, we compared the productions of IL‐6 and TGF‐β between DANCR‐expressing and vector MCF10A or MCF7 cells. As shown in Fig. 7A–D, both of their secretions into the conditioned medium of and the mRNA levels in MCF10A or MCF7 cells were strikingly induced by DANCR overexpression (*P* < 0.05, when compared with vector cells). On the molecular level, we found that DANCR expression potently activated NF‐κB and STAT3 (as represented by p‐p65 and p‐STAT3, respectively) but decreased SOCS3 level (*P* < 0.05, when compared with vector cells; Fig. 7E,F). The reduction in SOCS3 was associated with an increased level of H3K27me3 (Fig. 7G,H). CHIP‐qPCR assay showed the enhanced binding of the EZH2 or H3K27me3 to the promoter of SOCS3 [Fig. 7I,J; the overall binding was even lower than in the highly malignant MDA‐MB‐231 and MDA‐MB‐468 cells (Fig. 4I,J), as indicated by the percentage of input]. RIP assay showed an interaction between DANCR and EZH2 (Fig. 7K). These results suggest that in normal breast epithelial cells or breast cancer cells of low malignancy, overexpression of DANCR is capable of down‐regulating SOCS3 *via* interaction with EZH2, which is consistent with that of malignant cell lines.

### 3.8 Overexpression of SOCS3 or inhibition of EZH2 reversed the malignant phenotypes induced by elevating DANCR in normal breast epithelial cells or breast cancer cells of low malignancy

**Figure 8:**
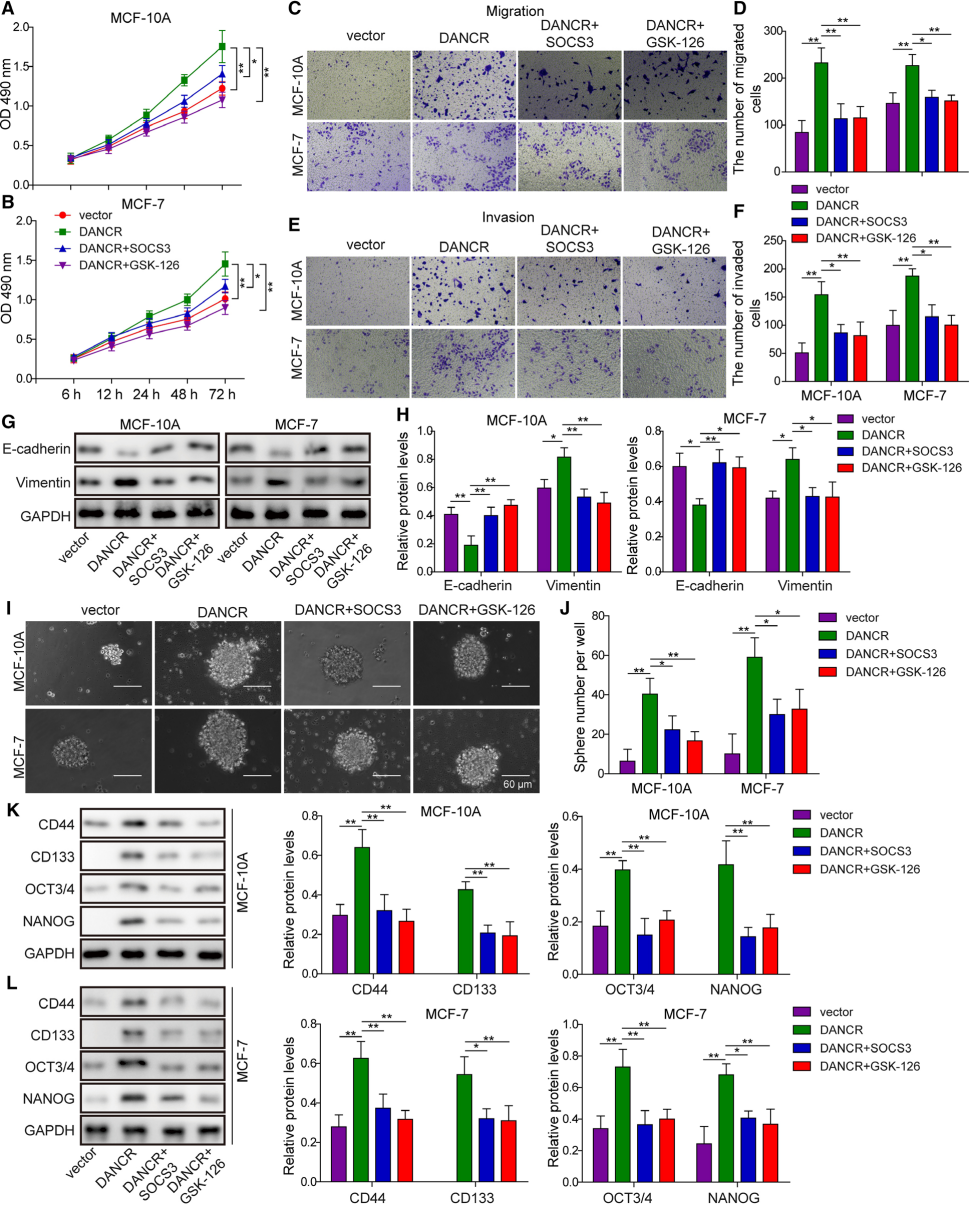
Overexpressing SOCS3 or inhibiting EZH2 rescued MCF‐10A or MCF‐7 cells from DANCR‐stimulated viability, migration, invasion, EMT or cancer stemness. (A,B) MTT assay showed that overexpressing SOCS3 in DANCR cells (DANCR + SOCS3) or treating these cells with EZH2 inhibitor (DANCR + GSK‐126) reduced DANCR‐stimulated cell viability. Transwell migration (C,D) and invasion (E,F) assay showed overexpression of SOCS3 or inhibition of EZH2 inhibited DANCR‐stimulated migration and invasion of indicated cells. (G,H) Western blot on EMT‐related biomarkers, E‐cadherin, and Vimentin showed that although DANCR stimulated EMT in MCF‐10A and MCF‐7 cells, overexpression of SOCS3 or treatment of cells with GSK‐126 was sufficient to abolish the effect of DANCR. Representative western images are shown in (G) and the quantification in (H). (I,J) Mammosphere assay showed that DANCR significantly promoted mammosphere formation, which was reversed by overexpression of SOCS3 or inhibition of EZH2. Representative images of mammosphere formed in each group are shown in (I) and the quantification on the number of mammospheres in (J). (K,L) Western blot showed DANCR increased the expression of stemness‐related biomarkers CD44, CD133, OCT3/4, and NANOG, which was inhibited by overexpressing SOCS3 or inhibiting EZH2. *n* = 3, data are shown as mean ± SD. One‐way ANOVA was used to determine statistical significance: *P < 0.05, **P < 0.01.

Suppression of cytokine signaling 3 was demonstrated to be a tumor suppressor in breast cancer (Barclay *et al.*, 2009). Our finding that DANCR negatively regulated the expression of SOCS3 through the epigenetic regulation by EZH2 and H3K27me3 suggests that down‐regulating SOCS3 may mediate the oncogenic activities of the former. To address the biological significance of SOCS3 and EZH2 in DANCR‐induced malignant phenotypes, we overexpressed SOCS3 (DANCR + SOCS3) or inhibited EZH2 (DANCR + GSK‐126) in DANCR‐expressing MCF10A or MCF7 cells. By monitoring the phenotypes of these cells, we found that overexpression of SOCS3 rescued multiple malignant phenotypes induced by DANCR in both MCF10A and MCF7 cells, including cell viability (Fig. 8A,B), migration (Fig. 8C,D), invasion (Fig. 8E,F), EMT (Fig. 8G,H), and cancer stemness (Fig. 8I–L). A similar rescue was also observed in DANCR + GSK‐126 cells.

**Figure 9:**
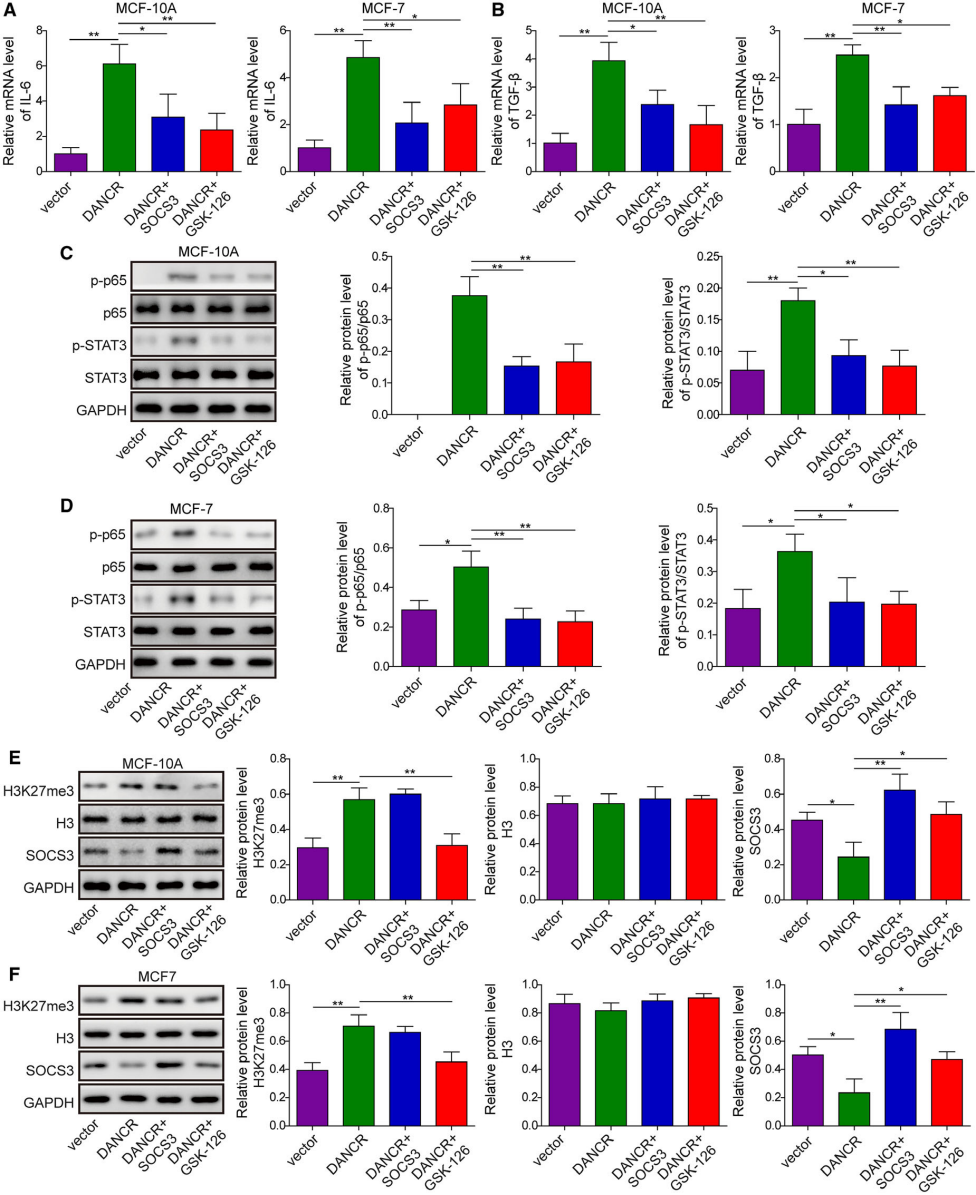
SOCS3 and EZH2 essentially mediated the effects of DANCR on inflammation. (A) qRT‐PCR showed that overexpression of SOCS3 or inhibition of EZH2 with GSK‐126 reduced DANCR‐stimulated mRNA of IL‐6 of indicated cells. (B) qRT‐PCR showed that overexpression of SOCS3 or inhibition of EZH2 with GSK‐126 reduced DANCR‐stimulated mRNA of TGF‐β of indicated cells. (C,D) Western blot showed that SOCS3 and GSK‐126 antagonized the effect of DANCR in activating p65 and STAT3. (E,F) Western blot on H3K27me3, H3, and SOCS3 showed that although overexpression of SOCS3 failed to alter the level of H3K27me3, whereas GSK‐126 significantly reduced it, both treatments significantly boosted the level of SOCS3 in DANCR cells. One‐way ANOVA was used to determine statistical significance: *P < 0.01, **P < 0.05.

On the molecular level, overexpression of SOCS3 or supplementation of GSK‐126 in DANCR overexpressed cells significantly reduced the expression of IL‐6 (Fig. 9A) and TGF‐β (Fig. 9B), and inhibited the activation of NF‐κB and STAT3 signaling (Fig. 9C,D). Correspondingly, although overexpressing SOCS3 failed to alter the level of H3K27me3, whereas GSK‐126 significantly reduced it, both treatments significantly elevated the level of SOCS3, suggesting that targeting SOCS3 is an essential mechanism by which DANCR could present pleiotropic oncogenic activities.

## 4 Discussion

In this study, we presented pleiotropic oncogenic activities of lncRNA DANCR on multiple levels. First, DANCR was up‐regulated in breast tissues (specifically in those associated with advanced pathological grades or positive lymph node metastasis) as well as in highly malignant and metastatic TNBC cells, supporting the clinical relevance of DANCR in breast cancer. Secondly, in TNBC cells, knocking down DANCR significantly reduced EMT, cancer stemness, and inflammation, suggesting its essential role in maintaining these malignant phenotypes of advanced breast cancer cells. Thirdly, in normal breast epithelial cells or breast cancer cells of low malignancy, overexpression of DANCR was sufficient to promote EMT, cancer stemness, and inflammation, indicating its potency in inducing tumorigenesis and malignant transformation during breast cancer oncogenesis. Lastly, on the molecular level, the activities of DANCR in normal, early‐ or late‐stage breast cancer cells were achieved by EZH2‐mediated epigenetic down‐regulation of SOCS3.

Cancer stemness, EMT, and inflammation are three closely interrelated mechanisms regulating the malignant phenotypes of cancer. Cancer stem cells are the driving force for cancer metastasis and recurrence, leading to more than 90% of breast cancer‐related death (Baccelli *et al.*, 2013; Peitzsch *et al.*, 2017). Phenotypically, breast CSC are identified by elevated expressions of stem‐cell biomarkers such as CD44, CD133, ALDH, OCT3/4, Nanog, and SOX2 (Ling *et al.*, 2012; Pattabiraman and Weinberg, 2014). Several reports showed that DANCR stimulates cancer stem‐cell features (Jiang *et al.*, 2017; Yuan *et al.*, 2016). For breast cancer, Sha *et al. *(2017) used MDA‐MB231 cells as the model system and demonstrated that knocking down DANCR was sufficient to reduce the expression of CSC markers, CD44, ALDH, and ABCG2, which correlated with inhibited xenograft formation *in vivo.* Consistent with Sha *et al*.’s findings, we showed that knocking down DANCR in TNBC cells potently reduced the endogenous expressions of CD44, CD133, OCT3/4, and Nanog, which was correlated with inhibited mammosphere formation *in vitro* and xenograft formation *in vivo* from shDANCR‐targeting TNBC cells. Furthermore, we showed for the first time that overexpressing DANCR in normal MCF10A or low‐malignant MCF7 cells significantly up‐regulated the expression of multiple CSC biomarkers, leading to increased mammosphere formation *in vitro* and stimulated tumor formation in mice, suggesting that DANCR not only was essential to maintain cancer stemness in late‐stage breast cancer cells but also was sufficient to transform normal breast epithelial cells into tumor‐forming cancer cells and to boost further the stemness of low‐malignant cancer cells.

Mechanistically, EMT is closely linked to cancer stemness (Wang *et al.*, 2015). The key EMT‐inducer TGF‐β is sufficient to generate CSC (Asiedu *et al.*, 2011; Shuang *et al.*, 2014). Although it is well demonstrated that DANCR stimulates the migration, invasion, and metastasis (all characteristic EMT phenotypes) of multiple human cancers (Jin *et al.*, 2017; Mao *et al.*, 2017; Pan *et al.*, 2018; Shi *et al.*, 2018; Wang and Jiang, 2018; Wang *et al.*, 2018a; 2018b), few studies have presented direct evidence that DANCR regulates EMT. In this study, we presented the correlation between high DANCR expression and tumors positive for lymph node metastasis. More importantly, we showed that knocking down endogenous DANCR significantly reduced the migration/invasion of TNBC cells, whereas overexpressing DANCR in normal MCF10A or low‐malignant MCF7 cells markedly stimulated their migration/invasion. The changes in cell migration/invasion were associated with alterations of EMT‐related biomarkers, including SNAIL1, SLUG, MMP‐2, MMP‐9, E‐cadherin, and Vimentin. These data demonstrate the causal relationship between DANCR and EMT in breast cancer and support the promotion cancer stemness by stimulation of EMT and DANCR.

Another mechanism critical for both EMT and cancer stemness is inflammation. Chronic inflammation, by activating two major signaling pathways, NFκB and STAT3, contributes critically to cancer initiation, progression, and metastasis (Blaylock, 2015). In this study, we focused on two inflammation‐related cytokines, TGF‐β and IL‐6. TGF‐β is a growth suppressor during normal breast development and shifts to a cytokine that essentially stimulates the motility, invasion, and metastasis of cancer cells during oncogenesis (Barcellos‐Hoff and Akhurst, 2009). The malignant conversion of TGF‐β is achieved by stimulating EMT and cancer stemness within cancer cells, inducing an immunosuppressive environment to minimize the elimination of cancer cells, and exacerbating the production of itself from cancer as well as other stromal cells (Barcellos‐Hoff and Akhurst, 2009). IL‐6 is another well‐demonstrated inducer of EMT and cancer stem features in breast cancer (Xie *et al.*, 2012). Yao *et al. *(2010) showed that TGF‐β, by activating IL‐6 production, not only induced EMT but provided a mechanism leading to resistance to erlotinib. In this study, we showed for the first time that DANCR essentially controlled the expression of both TGF‐β and IL‐6: shDANCR in TNBC cells significantly reduced their production, whereas DANCR overexpression in normal MCF10A or low‐malignant MCF7 cells potently stimulated it. The regulation on TGF‐β and IL‐6 was associated with the activation of NF‐κB and STAT3 signaling in different cells. These data suggest that elevation of TGF‐β and IL‐6 production is a mechanism by which DANCR promotes EMT and cancer stemness. They also further corroborate the intricate crosstalk among EMT, cancer stemness, and inflammation, and its role in promoting malignant development of cancer.

Cumulative evidence suggests the importance of the NF‐κB/STAT3/IL‐6 positive feedback loop in linking inflammation and cancer: to maintain the active state of breast myofibroblasts (Hendrayani *et al.*, 2016), to stimulate vascular inflammation (Brasier, 2010), and to promote the survival of cancer cells under stressed conditions and tumor aggressiveness (McFarland *et al.*, 2013; Yoon *et al.*, 2012). Concomitant with the activation of NF‐κB and STAT3 by DANCR overexpression or their deactivation by shDANCR in breast epithelial cells of different malignancies, we observed an opposing regulation between DANCR and SOCS3: by forming a complex with EZH2, DANCR promoted the binding of EZH2 to the promoter of SOCS3 and up‐regulated the level of H3K27me3, which collectively and epigenetically silenced the expression of SOCS3. SOCS3 is a negative regulator of cytokine signaling, such as IL‐6‐activated STAT3 (Wang *et al.*, 2013) or TGF‐β‐promoted Th17 cell development (Qin *et al.*, 2009). Dhar *et al. *(2013) showed that STAT3 and NFκB‐p65 interacted, leading to hypermethylation of SOCS3 promoter, and down‐regulated SOCS3 in human coronary artery smooth muscle cells. Kim *et al. *(2015) showed that in PTEN‐ and p53‐inactivated TNBC cells, SOCS3 inhibited IL‐6/STAT3/NFκB signaling. Considering that inflammation impacts not only cancer cells but also a variety of stromal cells, such as macrophages and fibroblasts, within the tumor microenvironment, it is interesting to explore the effects of DANCR on these cells and how such effects may provide feedback on the aggressive growth and metastasis of cancer cells.

## 5 Conclusions

In summary, we identified lncRNA DANCR as the master regulator of inflammation as well as inflammation‐mediated EMT and cancer stemness. DANCR not only essentially maintained these phenotypes in late‐stage TNBC but also sufficiently induced them in normal breast epithelial cells or early‐stage breast cancer cells. More importantly, we showed that the oncogenic activities of DANCR were achieved by EZH2‐mediated epigenetic down‐regulation of SOCS3. Therefore, understanding the mechanisms controlling DANCR expression during breast cancer development, effectively targeting these mechanisms, and developing strategies to either suppress DANCR or up‐regulate SOCS3 may prevent tumorigenesis and suppress the malignant progression of breast cancer.

## Conflict of interest

The authors declare no conflict of interest.

## Author contributions

LG conceived and designed the experiments. KJZ performed the experiments. Funding was acquired by LG. KJZ and XT contributed reagents/materials/analysis tools. XT wrote the paper. All authors read and approved the final manuscript.

## Supporting information


**Fig. S1.** DANCR was essential for maintaining cell‐cycle progression in highly malignant breast cancer cells. Cell‐cycle progression of shDANCR or shNC MDA‐MB‐231 (A) and MDA‐MB‐468 (B) was examined by PI staining followed by flow cytometry. The percentage of cells in indicated cell‐cycle phases was calculated as mean ± SD from three independent experiments and compared between shDANCR and shNC cells. Student’s *t‐*test was used to determine statistical significance: **P < 0.01.

**Fig. S2.** DANCR critically controlled lung metastasis *in vivo*. shDANCR or shNC MDA‐MB‐231 and MDA‐MB‐468 cells (A,B), DANCR‐overexpressing or vector MCF‐10A and MCF‐7 cells (C,D) were injected into mice through the tail vein (*n *= 5/group). At 5 weeks after the injection, all mice were sacrificed and the lung tissues were isolated. The number of surface metastases per lung was quantified under a dissecting microscope and averaged from all five mice of the same group. The representative lung images are shown in (A) and (C), and the number of metastatic nodules in (B) and (D). Student’s *t*‐test was used to determine statistical significance: *P < 0.05, **P < 0.01.

**Fig. S3.** DANCR persistently promoted the self‐renewal of CSC. Self‐renewal of CSC was examined in shNC vs. shDANCR MDA‐MB‐231 (A) or MDA‐MB‐468 (B), and vector vs. DANCR‐overexpressing MCF‐10A (C) and MFC‐7 (D) cells using mammosphere formation assay for up to four generations. The number of mammospheres is presented as mean ± SD from three independent experiments. Student’s *t*‐test was used to determine the statistical significance: *P < 0.05, **P < 0.01.

## References

[mol212622-bib-0001] Asiedu MK , Ingle JN , Behrens MD , Radisky DC and Knutson KL (2011) TGFbeta/TNF(alpha)‐mediated epithelial‐mesenchymal transition generates breast cancer stem cells with a claudin‐low phenotype. Cancer Res 71, 4707–4719. 21555371 10.1158/0008-5472.CAN-10-4554 PMC3129359

[mol212622-bib-0002] Baccelli I , Schneeweiss A , Riethdorf S , Stenzinger A , Schillert A , Vogel V , Klein C , Saini M , Bauerle T , Wallwiener M *et al* (2013) Identification of a population of blood circulating tumor cells from breast cancer patients that initiates metastasis in a xenograft assay. Nat Biotechnol 31, 539–544. 23609047 10.1038/nbt.2576

[mol212622-bib-0003] Barcellos‐Hoff MH and Akhurst RJ (2009) Transforming growth factor‐beta in breast cancer: too much, too late. Breast Cancer Res 11, 202. 19291273 10.1186/bcr2224 PMC2687712

[mol212622-bib-0004] Barclay JL , Anderson ST , Waters MJ and Curlewis JD (2009) SOCS3 as a tumor suppressor in breast cancer cells, and its regulation by PRL. Int J Cancer 124, 1756–1766. 19115200 10.1002/ijc.24172

[mol212622-bib-0005] Blaylock RL (2015) Cancer microenvironment, inflammation and cancer stem cells: A hypothesis for a paradigm change and new targets in cancer control. Surg Neurol Int 6, 92. 26097771 10.4103/2152-7806.157890 PMC4455122

[mol212622-bib-0006] Brasier AR (2010) The nuclear factor‐kappaB‐interleukin‐6 signalling pathway mediating vascular inflammation. Cardiovasc Res 86, 211–218. 20202975 10.1093/cvr/cvq076 PMC2912657

[mol212622-bib-0007] Chen L , Yao H , Wang K and Liu X (2017) Long non‐coding RNA MALAT1 regulates ZEB1 expression by sponging miR‐143‐3p and promotes hepatocellular carcinoma progression. J Cell Biochem 118, 4836–4843. 28543721 10.1002/jcb.26158

[mol212622-bib-0008] Dai X , Li T , Bai Z , Yang Y , Liu X , Zhan J and Shi B (2015) Breast cancer intrinsic subtype classification, clinical use and future trends. Am J Cancer Res 5, 2929–2943. 26693050 PMC4656721

[mol212622-bib-0009] Dhar K , Rakesh K , Pankajakshan D and Agrawal DK (2013) SOCS3 promotor hypermethylation and STAT3‐NF‐kappaB interaction downregulate SOCS3 expression in human coronary artery smooth muscle cells. Am J Physiol Heart Circ Physiol 304, H776–785. 23335796 10.1152/ajpheart.00570.2012 PMC3602771

[mol212622-bib-0010] Dou P , Hu R , Zhu W , Tang Q , Li D , Li H and Wang W (2017) Long non‐coding RNA HOTAIR promotes expression of ADAMTS‐5 in human osteoarthritic articular chondrocytes. Pharmazie 72, 113–117. 29441864 10.1691/ph.2017.6649

[mol212622-bib-0011] Fang Y and Fullwood MJ (2016) Roles, functions, and mechanisms of long non‐coding RNAs in cancer. Genomics Proteomics Bioinformatics 14, 42–54. 26883671 10.1016/j.gpb.2015.09.006 PMC4792843

[mol212622-bib-0012] Hendrayani SF , Al‐Harbi B , Al‐Ansari MM , Silva G and Aboussekhra A (2016) The inflammatory/cancer‐related IL‐6/STAT3/NF‐kappaB positive feedback loop includes AUF1 and maintains the active state of breast myofibroblasts. Oncotarget 7, 41974–41985. 27248826 10.18632/oncotarget.9633 PMC5173109

[mol212622-bib-0013] Hollier BG , Tinnirello AA , Werden SJ , Evans KW , Taube JH , Sarkar TR , Sphyris N , Shariati M , Kumar SV , Battula VL *et al* (2013) FOXC2 expression links epithelial‐mesenchymal transition and stem cell properties in breast cancer. Cancer Res 73, 1981–1992. 23378344 10.1158/0008-5472.CAN-12-2962 PMC3602160

[mol212622-bib-0014] Janssen LME , Ramsay EE , Logsdon CD and Overwijk WW (2017) The immune system in cancer metastasis: friend or foe? J Immunother Cancer 5, 79. 29037250 10.1186/s40425-017-0283-9 PMC5644253

[mol212622-bib-0015] Jia J , Li F , Tang XS , Xu S , Gao Y , Shi Q , Guo W , Wang X , He D and Guo P (2016) Long noncoding RNA DANCR promotes invasion of prostate cancer through epigenetically silencing expression of TIMP2/3. Oncotarget 7, 37868–37881. 27191265 10.18632/oncotarget.9350 PMC5122356

[mol212622-bib-0016] Jiang N , Wang X , Xie X , Liao Y , Liu N , Liu J , Miao N , Shen J and Peng T (2017) lncRNA DANCR promotes tumor progression and cancer stemness features in osteosarcoma by upregulating AXL via miR‐33a‐5p inhibition. Cancer Lett 405, 46–55. 28642170 10.1016/j.canlet.2017.06.009

[mol212622-bib-0017] Jin L , Fu H , Quan J , Pan X , He T , Hu J , Li Y , Li H , Yang Y , Ye J *et al* (2017) Overexpression of long non‐coding RNA differentiation antagonizing non‐protein coding RNA inhibits the proliferation, migration and invasion and promotes apoptosis of renal cell carcinoma. Mol Med Rep 16, 4463–4468. 28765964 10.3892/mmr.2017.7135

[mol212622-bib-0018] Katsuno Y , Lamouille S and Derynck R (2013) TGF‐beta signaling and epithelial‐mesenchymal transition in cancer progression. Curr Opin Oncol 25, 76–84. 23197193 10.1097/CCO.0b013e32835b6371

[mol212622-bib-0019] Kim G , Ouzounova M , Quraishi AA , Davis A , Tawakkol N , Clouthier SG , Malik F , Paulson AK , D'Angelo RC , Korkaya S *et al* (2015) SOCS3‐mediated regulation of inflammatory cytokines in PTEN and p53 inactivated triple negative breast cancer model. Oncogene 34, 671–680. 24531711 10.1038/onc.2014.4 PMC4285772

[mol212622-bib-0020] Ling GQ , Chen DB , Wang BQ and Zhang LS (2012) Expression of the pluripotency markers Oct3/4, Nanog and Sox2 in human breast cancer cell lines. Oncol Lett 4, 1264–1268. 23197999 10.3892/ol.2012.916 PMC3506717

[mol212622-bib-0021] Liu RY , Zeng Y , Lei Z , Wang L , Yang H , Liu Z , Zhao J and Zhang HT (2014) JAK/STAT3 signaling is required for TGF‐beta‐induced epithelial‐mesenchymal transition in lung cancer cells. Int J Oncol 44, 1643–1651. 24573038 10.3892/ijo.2014.2310

[mol212622-bib-0022] Livak KJ and Schmittgen TD (2001) Analysis of relative gene expression data using real‐time quantitative PCR and the 2(‐Delta Delta C(T)) Method. Methods 25, 402–408. 11846609 10.1006/meth.2001.1262

[mol212622-bib-0023] Lombardo Y , de Giorgio A , Coombes CR , Stebbing J and Castellano L (2015) Mammosphere formation assay from human breast cancer tissues and cell lines. J Vis Exp. Doi: 10.3791/52671. 10.3791/52671 PMC4401367 25867607

[mol212622-bib-0024] Mani SA , Guo W , Liao MJ , Eaton EN , Ayyanan A , Zhou AY , Brooks M , Reinhard F , Zhang CC , Shipitsin M *et al* (2008) The epithelial‐mesenchymal transition generates cells with properties of stem cells. Cell 133, 704–715. 18485877 10.1016/j.cell.2008.03.027 PMC2728032

[mol212622-bib-0025] Mao Z , Li H , Du B , Cui K , Xing Y , Zhao X and Zai S (2017) LncRNA DANCR promotes migration and invasion through suppression of lncRNA‐LET in gastric cancer cells. Biosci Rep 37: doi 10.1042/BSR20171070. 10.1042/BSR20171070 PMC5672085 28951520

[mol212622-bib-0026] McFarland BC , Hong SW , Rajbhandari R , Twitty GB Jr , Gray GK , Yu H , Benveniste EN and Nozell SE (2013) NF‐kappaB‐induced IL‐6 ensures STAT3 activation and tumor aggressiveness in glioblastoma. PLoS ONE 8, e78728. 24244348 10.1371/journal.pone.0078728 PMC3823708

[mol212622-bib-0027] Pan L , Liang W , Gu J , Zang X , Huang Z , Shi H , Chen J , Fu M , Zhang P , Xiao X *et al* (2018) Long noncoding RNA DANCR is activated by SALL4 and promotes the proliferation and invasion of gastric cancer cells. Oncotarget 9, 1915–1930. 29416741 10.18632/oncotarget.23019 PMC5788609

[mol212622-bib-0028] Pattabiraman DR and Weinberg RA (2014) Tackling the cancer stem cells ‐ what challenges do they pose? Nat Rev Drug Discov 13, 497–512. 24981363 10.1038/nrd4253 PMC4234172

[mol212622-bib-0029] Peitzsch C , Tyutyunnykova A , Pantel K and Dubrovska A (2017) Cancer stem cells: The root of tumor recurrence and metastases. Semin Cancer Biol 44, 10–24. 28257956 10.1016/j.semcancer.2017.02.011

[mol212622-bib-0030] Qian K , Liu G , Tang Z , Hu Y , Fang Y , Chen Z and Xu X (2017) The long non‐coding RNA NEAT1 interacted with miR‐101 modulates breast cancer growth by targeting EZH2. Arch Biochem Biophys 615, 1–9. 28034643 10.1016/j.abb.2016.12.011

[mol212622-bib-0031] Qin H , Wang L , Feng T , Elson CO , Niyongere SA , Lee SJ , Reynolds SL , Weaver CT , Roarty K , Serra R *et al* (2009) TGF‐beta promotes Th17 cell development through inhibition of SOCS3. J Immunol 183, 97–105. 19535626 10.4049/jimmunol.0801986 PMC2851540

[mol212622-bib-0032] Reiman JM , Knutson KL and Radisky DC (2010) Immune promotion of epithelial‐mesenchymal transition and generation of breast cancer stem cells. Cancer Res 70, 3005–3008. 20395197 10.1158/0008-5472.CAN-09-4041 PMC2856111

[mol212622-bib-0033] Sha S , Yuan D , Liu Y , Han B and Zhong N (2017) Targeting long non‐coding RNA DANCR inhibits triple negative breast cancer progression. Biol Open 6, 1310–1316. 28760736 10.1242/bio.023135 PMC5612229

[mol212622-bib-0034] Shi H , Shi J , Zhang Y , Guan C , Zhu J , Wang F , Xu M , Ju Q , Fang S and Jiang M (2018) Long non‐coding RNA DANCR promotes cell proliferation, migration, invasion and resistance to apoptosis in esophageal cancer. J Thorac Dis 10, 2573–2582. 29997918 10.21037/jtd.2018.04.109 PMC6006063

[mol212622-bib-0035] Shuang ZY , Wu WC , Xu J , Lin G , Liu YC , Lao XM , Zheng L and Li S (2014) Transforming growth factor‐beta1‐induced epithelial‐mesenchymal transition generates ALDH‐positive cells with stem cell properties in cholangiocarcinoma. Cancer Lett 354, 320–328. 25194504 10.1016/j.canlet.2014.08.030

[mol212622-bib-0036] Thin KZ , Liu X , Feng X , Raveendran S and Tu JC (2018) LncRNA‐DANCR: A valuable cancer related long non‐coding RNA for human cancers. Pathol Res Pract 214, 801–805. 29728310 10.1016/j.prp.2018.04.003

[mol212622-bib-0037] Torre LA , Bray F , Siegel RL , Ferlay J , Lortet‐Tieulent J and Jemal A (2015) Global cancer statistics, 2012. CA Cancer J Clin 65, 87–108. 25651787 10.3322/caac.21262

[mol212622-bib-0038] Velasco‐Velazquez MA , Popov VM , Lisanti MP and Pestell RG (2011) The role of breast cancer stem cells in metastasis and therapeutic implications. Am J Pathol 179, 2–11. 21640330 10.1016/j.ajpath.2011.03.005 PMC3123864

[mol212622-bib-0045] Wang Y and Zhou BP (2011) Epithelial‐mesenchymal transition in breast cancer progression and metastasis. Chin J Cancer 30, 603–611. 21880181 10.5732/cjc.011.10226 PMC3702729

[mol212622-bib-0041] Wang S and Jiang M (2018) The long non‐coding RNA‐DANCR exerts oncogenic functions in non‐small cell lung cancer via miR‐758‐3p. Biomed Pharmacother 103, 94–100. 29635134 10.1016/j.biopha.2018.03.053

[mol212622-bib-0040] Wang SS , Jiang J , Liang XH and Tang YL (2015) Links between cancer stem cells and epithelial‐mesenchymal transition. Onco Targets Ther 8, 2973–2980. 26527883 10.2147/OTT.S91863 PMC4621173

[mol212622-bib-0043] Wang Y , van Boxel‐Dezaire AH , Cheon H , Yang J and Stark GR (2013) STAT3 activation in response to IL‐6 is prolonged by the binding of IL‐6 receptor to EGF receptor. Proc Natl Acad Sci USA 110, 16975–16980. 24082147 10.1073/pnas.1315862110 PMC3801081

[mol212622-bib-0039] Wang R , Yan B , Li Z , Jiang Y , Mao C , Wang X and Zhou X (2017) Long non‐coding RNA HOX transcript antisense RNA promotes expression of 14‐3‐3sigma in non‐small cell lung cancer. Exp Ther Med 14, 4503–4508. 29067125 10.3892/etm.2017.5041 PMC5647736

[mol212622-bib-0042] Wang Y , Lu Z , Wang N , Feng J , Zhang J , Luan L , Zhao W and Zeng X (2018a) Long noncoding RNA DANCR promotes colorectal cancer proliferation and metastasis via miR‐577 sponging. Exp Mol Med 50, 57. 29717105 10.1038/s12276-018-0082-5 PMC5938019

[mol212622-bib-0044] Wang Y , Zeng X , Wang N , Zhao W , Zhang X , Teng S , Zhang Y and Lu Z (2018b) Long noncoding RNA DANCR, working as a competitive endogenous RNA, promotes ROCK1‐mediated proliferation and metastasis *via* decoying of miR‐335‐5p and miR‐1972 in osteosarcoma. Mol Cancer 17, 89. 29753317 10.1186/s12943-018-0837-6 PMC5948795

[mol212622-bib-0046] Xie G , Yao Q , Liu Y , Du S , Liu A , Guo Z , Sun A , Ruan J , Chen L , Ye C *et al* (2012) IL‐6‐induced epithelial‐mesenchymal transition promotes the generation of breast cancer stem‐like cells analogous to mammosphere cultures. Int J Oncol 40, 1171–1179. 22134360 10.3892/ijo.2011.1275 PMC3584811

[mol212622-bib-0047] Yao Z , Fenoglio S , Gao DC , Camiolo M , Stiles B , Lindsted T , Schlederer M , Johns C , Altorki N , Mittal V *et al* (2010) TGF‐beta IL‐6 axis mediates selective and adaptive mechanisms of resistance to molecular targeted therapy in lung cancer. Proc Natl Acad Sci USA 107, 15535–15540. 20713723 10.1073/pnas.1009472107 PMC2932568

[mol212622-bib-0048] Yoon S , Woo SU , Kang JH , Kim K , Shin HJ , Gwak HS , Park S and Chwae YJ (2012) NF‐kappaB and STAT3 cooperatively induce IL6 in starved cancer cells. Oncogene 31, 3467–3481. 22105366 10.1038/onc.2011.517

[mol212622-bib-0049] Yuan SX , Wang J , Yang F , Tao QF , Zhang J , Wang LL , Yang Y , Liu H , Wang ZG , Xu QG *et al* (2016) Long noncoding RNA DANCR increases stemness features of hepatocellular carcinoma by derepression of CTNNB1. Hepatology 63, 499–511. 25964079 10.1002/hep.27893

[mol212622-bib-0050] Zuo ZK , Gong Y , Chen XH , Ye F , Yin ZM , Gong QN and Huang JS (2017) TGFbeta1‐induced LncRNA UCA1 upregulation promotes gastric cancer invasion and migration. DNA Cell Biol 36, 159–167. 28075173 10.1089/dna.2016.3553

