# Supplementary figures and images for: The long non‐coding RNA DANCR regulates the inflammatory phenotype of breast cancer cells and promotes breast cancer progression via EZH2‐dependent suppression of SOCS3 transcription
Source: Mol Oncol. 2020 Jan 10;14(2):309–28. doi: 10.1002/1878-0261.12622 (PMC6998389; doi:10.1002/1878-0261.12622)

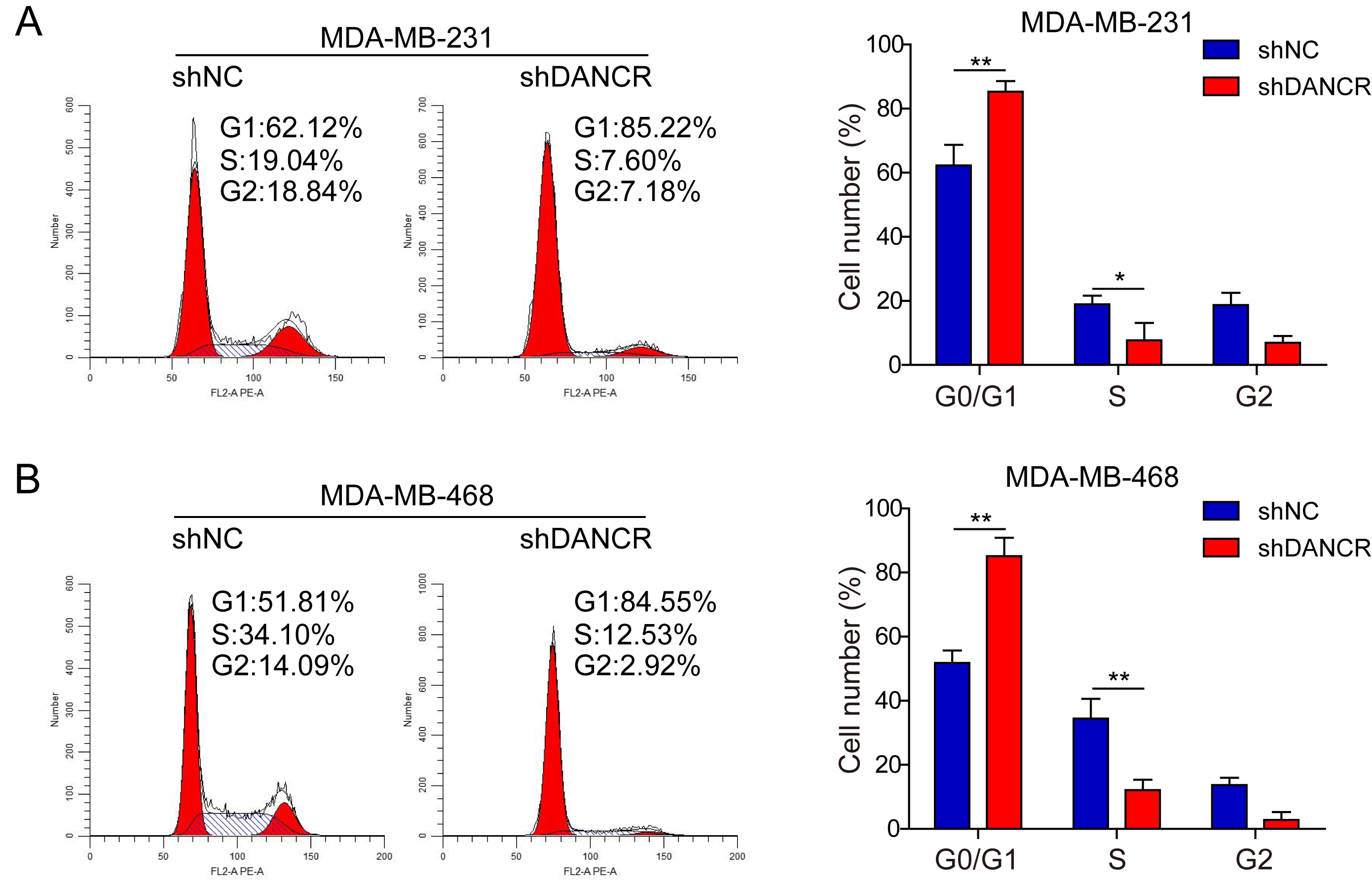

Supplement: Supplementary file 1 — Fig. S1. DANCR was essential for maintaining cell‐cycle progression in highly malignant breast cancer cells. Cell‐cycle progression of shDANCR or shNC MDA‐MB‐231 (A) and MDA‐MB‐468 (B) was examined by PI staining followed by flow cytometry. The percentage of cells in indicated cell‐cycle phases was calculated as mean ± SD from three independent experiments and compared between shDANCR and shNC cells. Student’s t‐test was used to determine statistical significance: **P < 0.01. [file MOL2-14-309-s001.jpg]

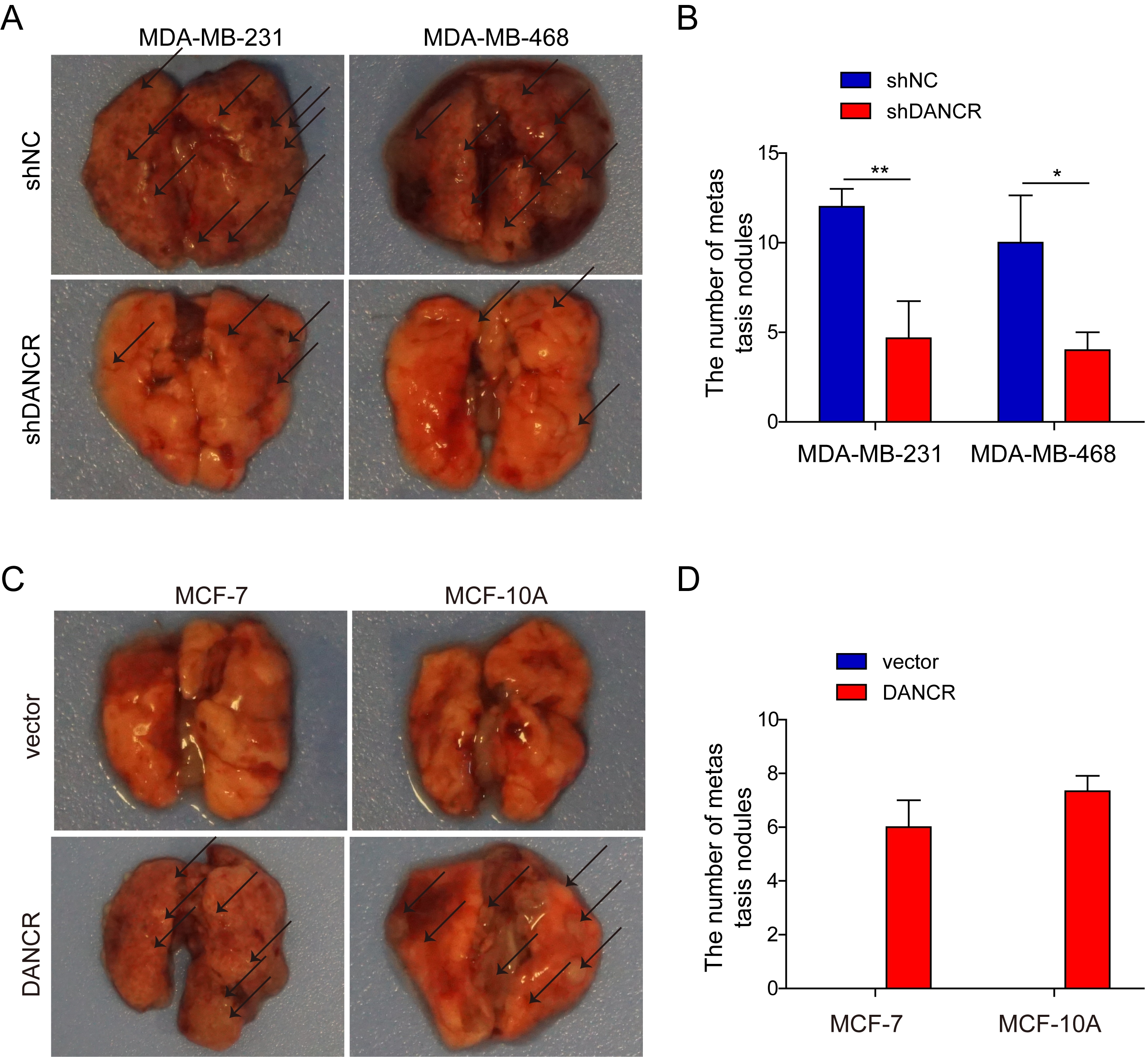

Supplement: Supplementary file 2 — Fig. S2. DANCR critically controlled lung metastasis in vivo. shDANCR or shNC MDA‐MB‐231 and MDA‐MB‐468 cells (A,B), DANCR‐overexpressing or vector MCF‐10A and MCF‐7 cells (C,D) were injected into mice through the tail vein (n = 5/group). At 5 weeks after the injection, all mice were sacrificed and the lung tissues were isolated. The number of surface metastases per lung was quantified under a dissecting microscope and averaged from all five mice of the same group. The representative lung images are shown in (A) and (C), and the number of metastatic nodules in (B) and (D). Student’s t‐test was used to determine statistical significance: *P < 0.05, **P < 0.01. [file MOL2-14-309-s002.jpg]

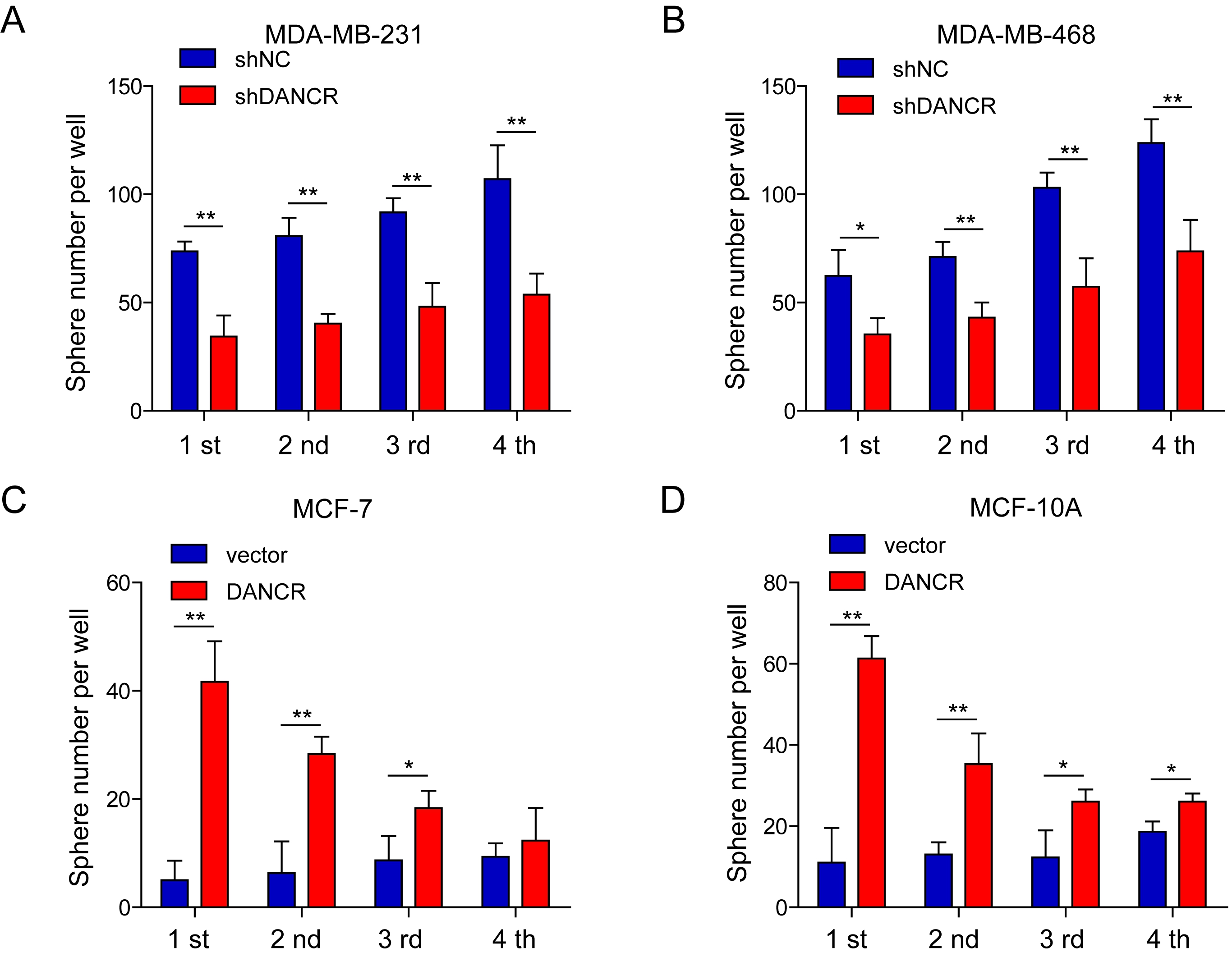

Supplement: Supplementary file 3 — Fig. S3. DANCR persistently promoted the self‐renewal of CSC. Self‐renewal of CSC was examined in shNC vs. shDANCR MDA‐MB‐231 (A) or MDA‐MB‐468 (B), and vector vs. DANCR‐overexpressing MCF‐10A (C) and MFC‐7 (D) cells using mammosphere formation assay for up to four generations. The number of mammospheres is presented as mean ± SD from three independent experiments. Student’s t‐test was used to determine the statistical significance: *P < 0.05, **P < 0.01. [file MOL2-14-309-s003.jpg]
